# Internet of Robotic Things Intelligent Connectivity and Platforms

**DOI:** 10.3389/frobt.2020.00104

**Published:** 2020-09-25

**Authors:** Ovidiu Vermesan, Roy Bahr, Marco Ottella, Martin Serrano, Tore Karlsen, Terje Wahlstrøm, Hans Erik Sand, Meghashyam Ashwathnarayan, Micaela Troglia Gamba

**Affiliations:** ^1^SINTEF Digital AS, Oslo, Norway; ^2^Infineon Technologies Austria AG, Villach, Austria; ^3^Insight Centre for Data Analytics, National University of Ireland Galway, Galway, Ireland; ^4^NxTech AS, Fredrikstad, Norway; ^5^Infineon Technologies India Pvt. Ltd., Bangalore, India; ^6^CISC Semiconductors GmbH, Klagenfurt am Woertersee, Austria

**Keywords:** internet of robotic things, internet of things, industrial internet of things, cyber-physical systems, collaborative robotics, distributed architectures, interoperability, trustworthiness

## Abstract

The Internet of Things (IoT) and Industrial IoT (IIoT) have developed rapidly in the past few years, as both the Internet and “things” have evolved significantly. “Things” now range from simple Radio Frequency Identification (RFID) devices to smart wireless sensors, intelligent wireless sensors and actuators, robotic things, and autonomous vehicles operating in consumer, business, and industrial environments. The emergence of “intelligent things” (static or mobile) in collaborative autonomous fleets requires new architectures, connectivity paradigms, trustworthiness frameworks, and platforms for the integration of applications across different business and industrial domains. These new applications accelerate the development of autonomous system design paradigms and the proliferation of the Internet of Robotic Things (IoRT). In IoRT, collaborative robotic things can communicate with other things, learn autonomously, interact safely with the environment, humans and other things, and gain qualities like self-maintenance, self-awareness, self-healing, and fail-operational behavior. IoRT applications can make use of the individual, collaborative, and collective intelligence of robotic things, as well as information from the infrastructure and operating context to plan, implement and accomplish tasks under different environmental conditions and uncertainties. The continuous, real-time interaction with the environment makes perception, location, communication, cognition, computation, connectivity, propulsion, and integration of federated IoRT and digital platforms important components of new-generation IoRT applications. This paper reviews the taxonomy of the IoRT, emphasizing the IoRT intelligent connectivity, architectures, interoperability, and trustworthiness framework, and surveys the technologies that enable the application of the IoRT across different domains to perform missions more efficiently, productively, and completely. The aim is to provide a novel perspective on the IoRT that involves communication among robotic things and humans and highlights the convergence of several technologies and interactions between different taxonomies used in the literature.

## Introduction

The IoRT enables robotic things in different environments to become active participants in various applications and exchange/share information with other robotic things, IoT/IIoT devices and humans. Robotic things are capable of recognizing events and changes in their surroundings while autonomously acting and reacting appropriately. These capabilities enable the convergence of the real, digital, virtual, cyber attributes of robotic things, and the creation of smart environments that make robotic things in the energy, mobility, buildings, manufacturing, and other sectors more intelligent.

Robotic engineering systems are deployed today in industry and are considered vital elements for the progress of humanity from an industrial perspective in the new digital age. As technologies such as IIoT, AI, robotics, intelligent connectivity, and electric mobility evolve, these systems are transformed in industrial IoRT applications.

New developments in intelligent connectivity enable robotic things to be connected at any time, in any place, and with anything and anyone through different paths/networks and services. In the future, an intelligent network infrastructure that is dynamically enhanced and extended by edge nodes, which are generated by interconnected robotic things, could serve as the backbone for IoRT applications.

The IoRT combines autonomous robotic systems with the IoT/IIoT, intelligent connectivity, distributed and federated edge/cloud computing, Artificial Intelligence (AI), Digital Twins (DT), Distributed Ledger Technologies (DLTs), Virtual/Augmented Reality (VR/AR), and swarm technologies. These technologies allow uniquely addressable intelligent things to interact and communicate with each other over the Internet and via other connectivity network protocols. The rapid development and deployment of multi-radio access technologies to allow devices and things to connect/interact at the edge of the IoRT have generated the development of heterogeneous mobile networks with a complex configuration that requires advanced device management and maintenance to cope with future robotic things (Vermesan et al., 2017a).

The convergence of IoT/IIoT, AI and robotics accelerates IoRT applications development, which improves the contextually aware decision-making support for resolving complex operations and enabling machine intelligence. This trend allows the convergence of programming systems, tools and controls, the use of core semantic web technologies and the interaction with robotic things to be implemented more efficiently.

**Figure 1:**
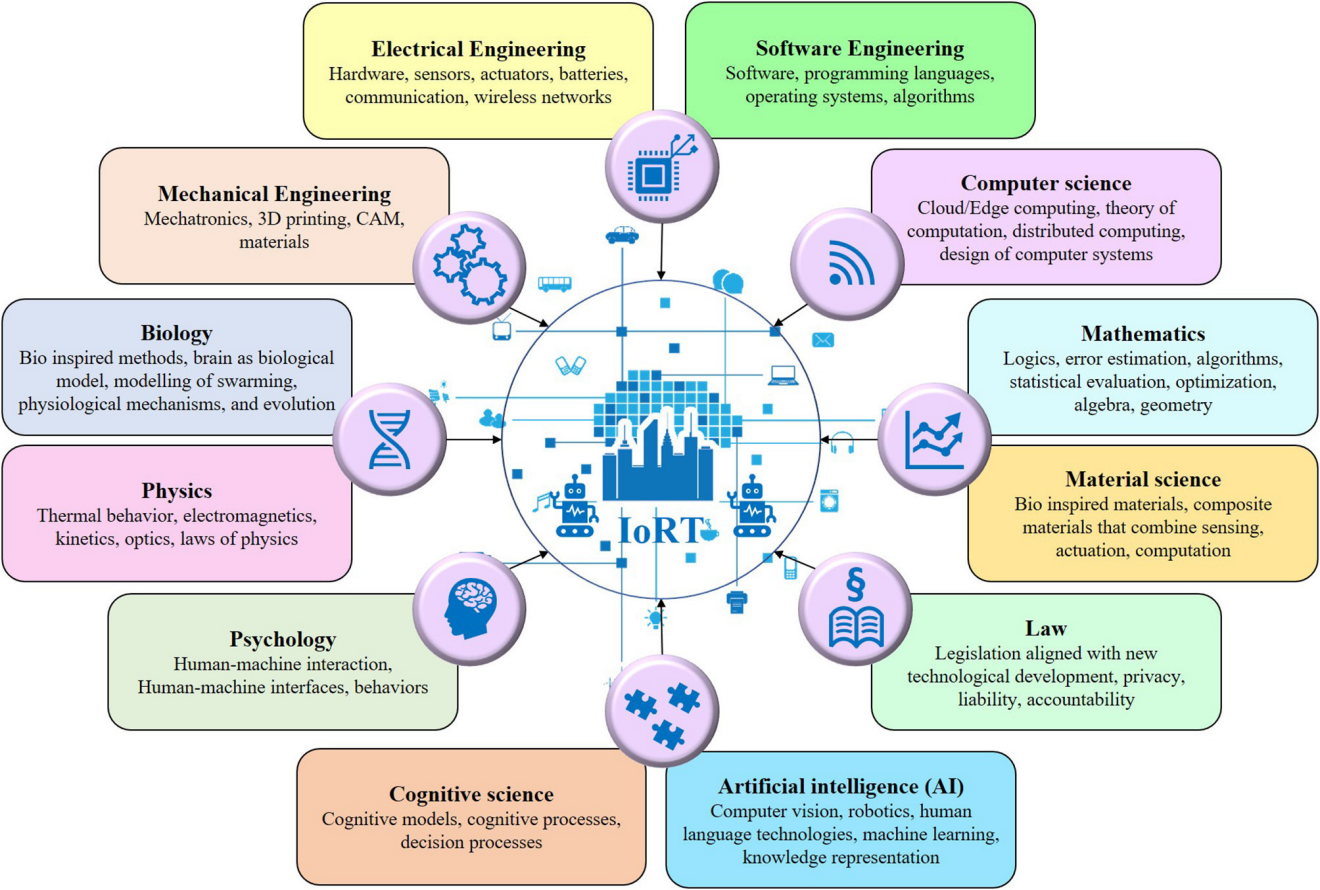
IoRT - An interdisciplinary branch of engineering and science.

Traditionally, robotics systems include a programmable dimension that is designed for repetitive, labor-intensive work, including sensing, and acting upon an environment (Vermesan et al., 2017b). The emergence of AI and Machine Learning (ML) has allowed robotic things to function using learning algorithms and cognitive decision-making rather than traditional programming. Combining different branches and scientific disciplines (Figure 1) makes it possible to develop autonomous programmable systems that combine robotics and machine learning. The IoRT multidisciplinary nature brings various perspectives from different disciplines and offers interdisciplinary solutions that consider the reciprocal effects and interactions between the multiple dimensions of next-generation IoRT ecosystems.

This paper is intended for researchers and developers engaged in the areas of IoT/IIoT, robotics, AI, DLTs, communication, software technologies and is organized as follows. Section Introduction presents the topic by providing background information on the research and innovation in IoRT. Section Internet of Robotic Things Taxonomy discusses the concept and the definition of IoRT used in the paper, along with the IoRT taxonomy. Section Enabling Technologies highlights the technologies enabling IoRT emphasizing the challenges of the convergence of these technologies in IoRT developments. The IoRT 3D architectural approach is presented in section IoRT Architectural Approach. The approach extends the current IoRT architectures described in the literature and introduces the evolution from centralized to decentralized and distributed architecture. The intelligent connectivity technologies for IoRT applications are described in section Intelligent Connectivity. An overview of the requirements and challenges for IoRT platforms and interoperability issues are introduced in sections IoRT Platforms and Interoperability in IoRT. The concept of IoRT technology and application trustworthiness is included in section Trustworthiness in IoRT with a description of the system properties need to assure IoRT system dependability and end-to-end and by-design/by-default properties/functionalities. Section IoRT Applications introduces several examples of emerging IoRT applications, while section Open issues and Future Directions of Research addresses the future research challenges. The synopsis and concluding remarks are drawn in section Summary and Conclusions by highlighting the leading technologies driving the IoRT developments.

## Internet of Robotic Things Taxonomy

The next-generation Internet landscape is expanding, and with it, the IoT/IIoT technologies and applications. IoT/IIoT devices become intelligent, mobile, autonomous, and operate in various environments, connect with other IoT/IIoT heterogeneous devices and are part of different applications in various industrial sectors and across the industries. As these technologies reach different application sectors and due to specific use in these domains (Vermesan et al., 2017b; Simoens et al., 2018), the original IoT/IIoT paradigm is evolving, expanding, and heading to significant developments in terms of research and innovation. New terminologies and concepts such as the Cognitive Internet of Things (Wu et al., 2014), the Internet of Mobile Things (IoMBT), the Autonomous Internet of Things (A-IoT), the Autonomous System of Things (ASoT), Internet of Underwater Things (IoUT), Internet of Drone Things (IoDT) (Nayyar et al., 2020), Internet of Nano Things (IoNT) (Nayyar et al., 2017), the Internet of Autonomous Things (IoAT), the Internet of Things Clouds (IoT-C), Internet of Cloud Things (IoCT) (Saha and Dasgupta, 2018), Mobile Cloud Robotics, Web of Robotic Things (WoRT) (Grieco et al., 2014), and Cloud Robotics are emerging and applied in different applications.

IoRT is associated with technological convergence challenges and opportunities that call for solutions to be addressed in the future (Ray, 2016; Vermesan et al., 2017b; Simoens et al., 2018). Various technologies are needed for ensuring the connectivity and programmability of multiple heterogeneous robotic things to implement cooperation/coordination functions, system configuration, information exchange, system dependability, and privacy. Developments in heterogeneous IoRT processing and dynamic autonomous systems build on decentralized architectures, parallelism and concurrency require new concepts for integrating intelligent, cooperative, and collaborative robotic things with other IoT/IIoT applications. It is essential to consider dynamic dependability, self-healing, resource self-repair, changing resource states, configuration/reconfiguration, real-time over-the-air (OTA) updates, device orchestration, and context-based/context-aware IoRT systems for service implementation and integration into the IoRT network. Furthermore, new “cognitive” robotic devices are being integrated into other IoT/IIoT applications and are becoming active participants in these applications, considering the context in which they are operating and interacting.

There is no standard definition for the “context” of IoRT applications. In this article, the definition provided by Dey et al. (2001) is used, stating that context represents “any information that can be used to characterize the situation of an entity” (e.g., a person, place, or object/thing that is identified relevant to the interaction). Context categories include location, status, time, identity. The location represents the geographical or spatial place attributes, the status reflects the intrinsic features of the elements in the context, time aligns the events and their status change in chronological order, and the identity assigns a unique identifier to an entity/object/thing to differentiate each entity and context.

The IoRT concept is derived from the IoT/IIoT paradigm. It applies to intelligent autonomous robotic things that are part of network infrastructures, with specific capabilities (sensing, actuation, processing, cognition, manipulation, motion, communication, mobility, population, autonomy, etc.), which collaborate/interact in a distributed manner to enable services and applications in different and across industrial domains.

As the concept of IoRT is evolving and expanding, there are many definitions proposed in the literature (Kara and Carlaw, 2014; Ray, 2016; Vermesan et al., 2017b; Simoens et al., 2018). The definition proposed by Kara and Carlaw (2014), states that IoRT is characterized by “intelligent devices that can monitor events, fuse sensor data from a variety of sources, use local and distributed intelligence to determine the best course of action,” while the definition provided by Ray (2016) focuses more on the robotic cloud concept.

A definition of IoRT needs to combine the existing definitions of IoT/IIoT with the terminology used in autonomous systems, collaborative robotics, distributed processing systems, AI, DT, edge/cloud computing, and DLTs. In this context, the definition of the robot provided by ISO 8373 (2012), which make the distinction between application areas of robotics (e.g., industrial and service domains), states that a robot is a “programmed actuated mechanism with a degree of autonomy, moving within its environment, to perform intended tasks.” The same standard defines the autonomy as “ability to perform intended tasks based on current state and sensing, without human intervention.” The IEEE 1872 (2015) standard offers a common set of term definitions, to facilitate the knowledge transfer unambiguously among groups of humans, robots, autonomous systems, and other artificial systems. The standard defines the robot as “an agentive device purposed to act in the physical world in order to accomplish one or more tasks…,” and in some instances, “the actions of a robot might be subordinated to actions of other agents, such as software agents (bots) or humans. A robot is composed of suitable mechanical and electronic parts. Robots might form social groups, where they interact to achieve a common goal. A robot (or a group of robots) can form robotic systems together with special environments geared to facilitate their work.”

In this paper, the IoRT is defined as a “dynamic global network infrastructure with self-configuring capabilities based on standard and interoperable communication protocols where physical and virtual (digital twins) “robotic things” have different degrees of mobility, autonomy, perception, actuation, identities, physical attributes, and virtual personalities, use intelligent interfaces, perception, processing, propulsion, cognition and connectivity, to take decisions and act based on real-time context conditions, interact, collaborate with other “things,” virtual and digital agents (bots) in various contexts, environments, and seamlessly use the information network to enable advanced secure, safe, trustworthy applications, services to achieve a common goal.” The definition is based on the existing IoT definition (Vermesan et al., 2011), and the terminology used in autonomous systems, collaborative robotics, distributed processing systems, and AI.

**Figure 2:**
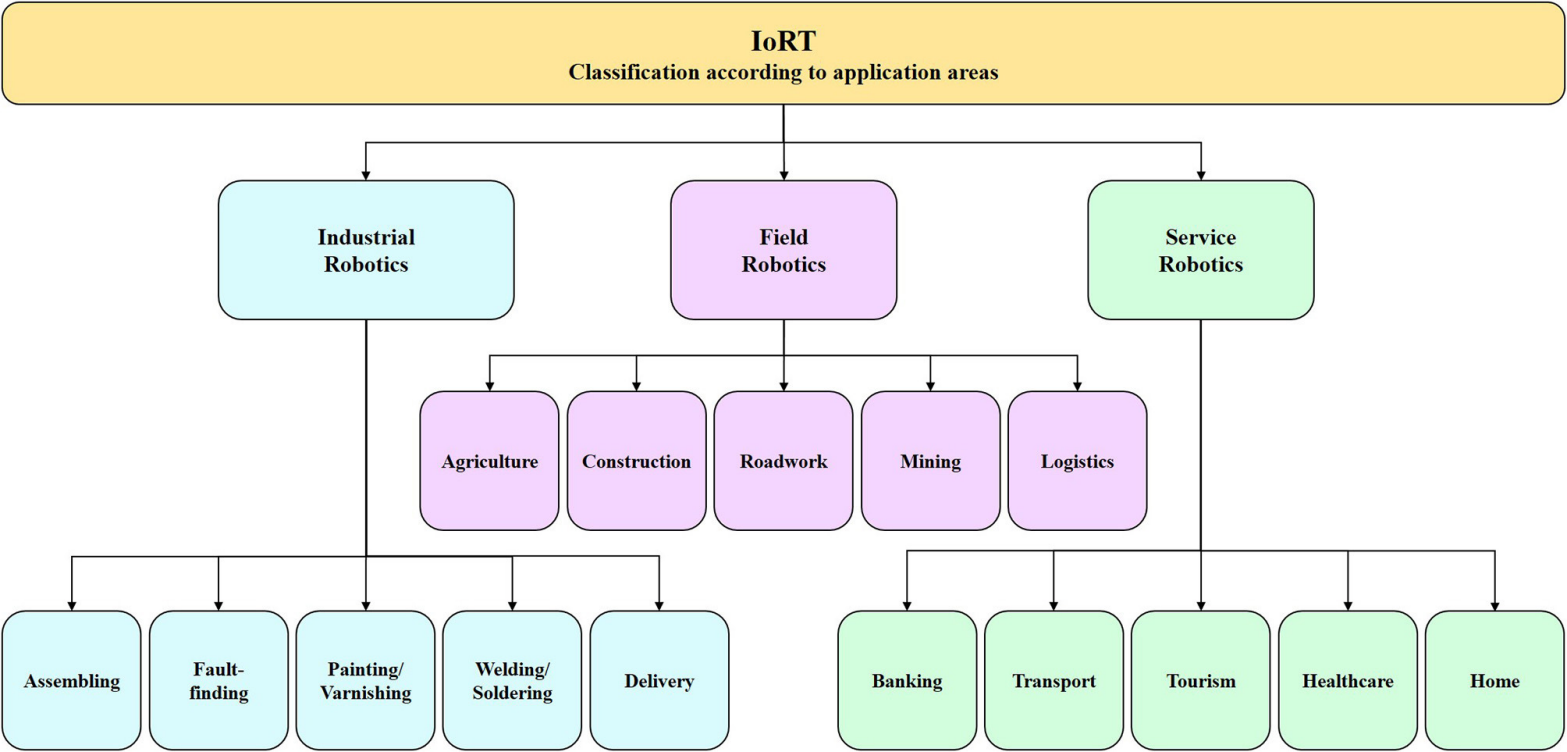
IoRT classification according to application areas.

Following the distinction between requirements and features of IoRT, a classification of the robotic things according to application areas is presented in Figure 2. The application areas presented are aligned with the description defined by ISO 8373 (2012) (e.g., industrial and service domains).

Other standards, such as ISO 10218-1 (2011) specifies requirements and guidelines for the inherent safe design, protective measures and information for the use of industrial robots by describing basic hazards associated with robots and providing requirements to eliminate, or adequately reduce, the risks associated with these hazards. In addition, ISO 10218-2 (2011) specifies safety requirements for the integration of industrial robots, industrial robot systems (as defined in ISO 10218-1), and industrial robot cell(s) by describing the basic hazards and hazardous situations identified for robotic systems, and provides the requirements to eliminate or adequately reduce the risks associated with these hazards. The IEEE 1872.1 (2017) standard is a logical extension to IEEE 1872 (2015) that extends the CORA ontology by defining additional ontologies appropriate for Autonomous Robotics (AuR). The (IEEE 1872.2, 2017) standard defines an ontology that allows for the representation of, reasoning about, and communication of task knowledge in the robotics and automation domain.

The standardization activities in ISO TC184—Automation systems and integration SC2 committee were organized under different working groups with the following focus: WG1 addressing all definitions in ISO 8373, WG3 focusing on industrial robot safety and WG7 addressing personal care safety. WG8 is coordinating the work of the other working groups within the service robotics area and determines the need for additional standards in the non-industrial robotics sector.

ISO/TC 184/SC 2 was upgraded to ISO/TC 299 with the title of “Robotics” in 2016. These changes over the years have reflected the increasing and broadening standardization activities in the field of robotics. The activities are carried on under WG1—Vocabulary and characteristics (ISO 9787, ISO 19649, ISO 8373), WG 2—Personal care robot safety (ISO 13482, ISO/CD TR 23482-1, ISO/CD TR 23482-2), WG 3—Industrial safety (ISO 10218-1, ISO 10218-2, ISO/TS15066), WG 4—Service robots (ISO 18646-1, ISO 18646-2, ISO/DIS 18646-3), JWG 5—Medical robot safety (IEC/TR 60601-4-1, IEC 80601-2-77, IEC 80601-2-78), and WG6—Modularity for service robots (ISO WD 22166-1).

**Figure 3:**
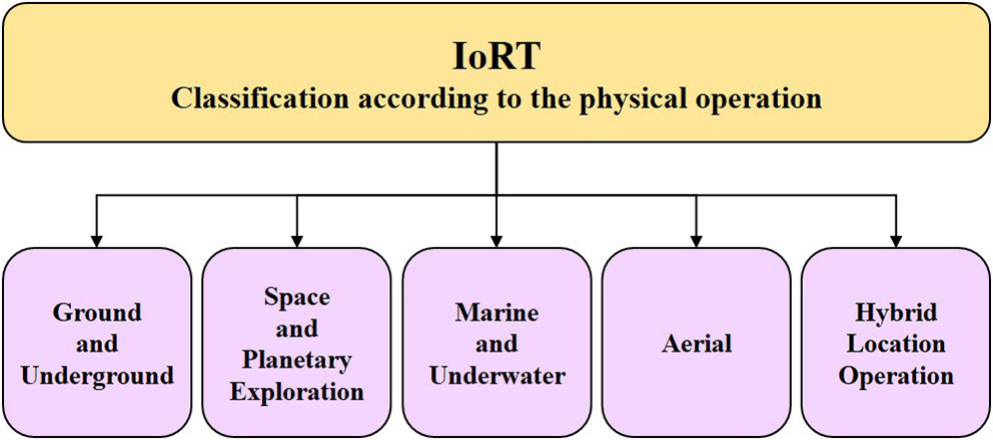
IoRT classification according to the physical operation.

The IoRT technologies and applications are developing considering the environmental conditions and the spatial context in which the IoRT devices are operating. Based on this consideration, the IoRT applications are classified as presented in Figure 3.

**Figure 4:**
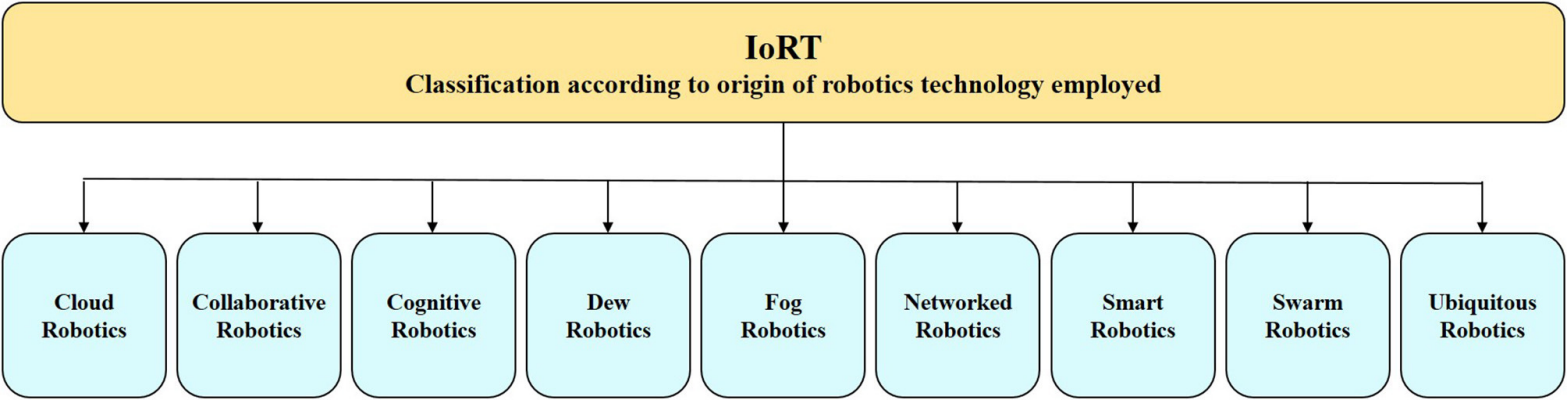
IoRT classification according to origin of robotics technology employed.

A different IoRT taxonomy is based on the origin of robotics technology employed for the different applications, as presented in Figure 4.

The robotics technologies highlighted in Figure 4 have specific characteristics that can be integrated into the IoRT developments and deployments. The short description of these characteristics is provided below.

Cloud robotics is defined as a cloud-centric technology where the “robots are connected to cloud computing infrastructure” to get access “to distributed computing resources” with “the ability to share training and labeling data for robot learning” (Jordan et al., 2013; Saha and Dasgupta, 2018).

Collaborative robotics (co-bot) is defined as a technology where the robot is designed and programmed to physically interact with humans in a commonly used environment and workspace (Popovic, 2013). The example applies to industrial robots/manipulators capable of operating safely in a common human-robot space and context.

Cognitive robotics is considered a technology that allows enabling “a robot with intelligent behavior by providing it with a processing architecture that will allow it to learn and reason about how to behave in response to complex goals in a complex world” (Liu et al., 2017).

Dew robotics technology is linked to “dew computing” considering the tasks “extremely distributed” over many machines, “which are heterogeneous, *ad-hoc* programmable, and self-adaptive.” Dew computing does not require the use of central nodes for implementing distributed applications. “The emphasis is on the architecture and the use of the resources available on the ground” (Botta et al., 2019).

Fog robotics is addressing technology that is based on robot systems that use fog computing for processing data and services (Gudi et al., 2019).

Networked robotics is addressing technology that includes “multiple robots operating together coordinating and cooperating by networked communication to accomplish a specified task” (Kumar et al., 2008).

Smart robotics is referring to technology using “an embodied AI system that can learn from its environment and its experience and build on its capabilities based on that knowledge” (Murphy, 2000).

Swarm robotics is using a technology based on “an approach to the coordination of multiple robots as a system which consists of large numbers of mostly simple physical robots” (Tan and Zheng, 2013).

Ubiquitous robotics is addressing the technology for “integrating robotic technologies with technologies from the fields of ubiquitous and pervasive computing, sensor networks, and ambient intelligence” (Kim et al., 2007).

**Figure 5:**
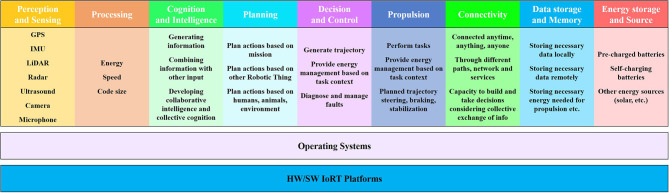
IoRT functional blocks.

The essential characteristics and functional blocks of IoRT systems for all applications and operating conditions are based on several fundamental principles inherited from the IoT/IIoT, robotic systems, AI, and intelligent connectivity, as described below and illustrated in Figure 5:

- Perception and sense—the ability of the IoRT system to sense the environment using different sensor types (e.g., microphones, ultrasound, radar, LiDAR, cameras, antennas) (Berger Roland Strategy Consultants, 2014), fuse the information from the different sensors and localize itself and other things, objects (Guo et al., 2019), humans, and animals using GPS/GNSS signals and both local and high-definition maps to create a semantic understanding and local and world models. Perception information is the input to analytics and AI processing, and the data collected by perception devices must be of a form suitable to be used by different and distinct cognitive processes at the robotic things and applications levels.
- Processing—the function used for optimal processing of information at the local, edge and cloud levels, more efficient data processing algorithms (energy, speed, code size, etc.) integrated into robotic things and across the distributed environments.
- Cognition and intelligence—the function used for generating information and combining that information with sensor and contextual inputs to generate intelligence in the form of decisions or knowledge to control the system's operations.
- Planning—the ability to plan actions based on the mission, fleet activities and information received from other robotic things, humans, animals, the environment, fleet managers, etc.
- Decision and control—the function of the IoRT system to generate a trajectory, choose a direction, act by sensing/actuating/moving/manipulating, provide energy management based on the task and context, diagnose and manage faults and engage in reactive control.
- Propulsion—the ability of the IoRT system to perform tasks, to move according to the environment (static or dynamic) of the thing in coordinated space and to control that movement based on the surrounding conditions—as defined by the safe operations in the collective system and fleets—by executing the planned trajectory using steering, body movements, braking and body stabilization.
- Connectivity—the ability of the IoRT system to be connected in any place, at any time, with anything and anyone using various paths/networks and services, thereby allowing the necessary level of autonomy and the capacity to build and make decisions considering the collective exchange of information among robotic things, humans, infrastructure and other IoT/IIoT applications.
- Storage of data/information/knowledge and energy—the function used for storing the necessary data/information/knowledge locally (memory) and remotely (edge, cloud, other robotic things, infrastructure) and the energy needed for propulsion (e.g., batteries for self-charging or as a source of pre-charged energy).

Digital transformation of society accelerates the development of applications in which IoRT assist, improve and minimize the load for human activities, and robotic engineering systems support further developments to optimize in an intelligent manner different humans-machines labor tasks. The emergence of intelligent systems, things, and their applications—in the form of collaborative IoRT autonomous fleets—requires new architectures, connectivity paradigms, and trustworthiness frameworks for the deployment of applications across different business and industrial domains.

This evolution toward more advanced edge computing and distributed machine learning is driven by several requirements for IoRT applications: real-time performance, reliable low-latency communication, energy efficiency, security/privacy of data/information, cognitive and collective collaborative behaviors.

The new IoRT applications accelerate the convergence between the development of more autonomous and intelligent system design paradigms and the IoT, in which collaborative robotic things can communicate with other “things,” learn autonomously, interact safely with the environment, humans and other things, and gain qualities such as self-maintenance, self-awareness, self-healing and fail-operational behavior. IoRT applications can make use of the individual, collaborative, and collective intelligence of robotic things, as well as information from the infrastructure and operating context, to plan and implement tasks in different environmental conditions, considering uncertainties and critical situations.

IoRT applications are developing alongside advancements in the IIoT, combining information technologies (IT) used for data-centric computing and operational technologies (OT) used in enterprise and industrial operations integrating supervisory control and data acquisition (SCADA) and programmable logic controllers (PLCs) systems, where the industrial applications are increasingly more integrated, and new intelligent connectivity networks are used. These applications can include heterogeneous and distributed IoRT applications that expose these connectivity networks to various and specific requirements. These new intelligent connectivity networks can deliver multiple functionalities and adaptive features that implement components of IoRT platforms and transfer information that meets IoRT applications' requirements in terms of content and context. IoRT applications can use network-generated data, and these multiple functionalities and adaptive features, which operate in real-time, can be dynamically initiated near IoRT fleets where data is generated, needed, and used.

**Figure 6:**
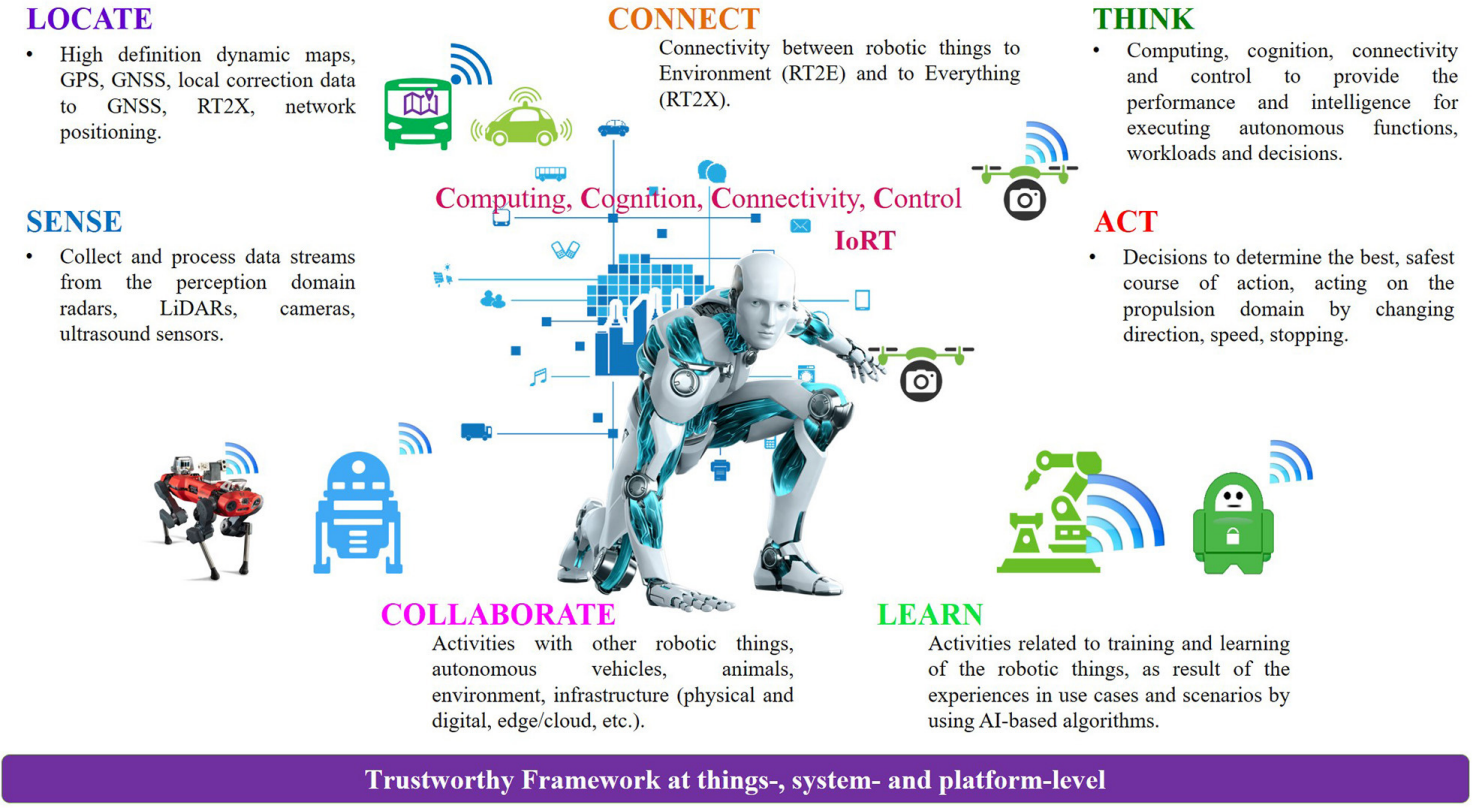
Autonomous and connected IoRT systems functions.

The entire digital value chain of future autonomous and connected IoRT systems must be able to SENSE, LOCATE, THINK, CONNECT, COLLABORATE, LEARN, and ACT as illustrated in Figure 6.

Therefore, the autonomous systems paradigm SENSE, LOCATE, THINK, CONNECT, COLLABORATE, LEARN, and ACT is increasingly being adopted, which could result in a paradigm shift in IoRT architecture, associated components, and the whole approach toward software, hardware, algorithms and the use of AI techniques and methods. It addresses the cognition, computing, control, connectivity for sensing, detection, perception, processing, decision functions so that the IoRT devices see and interpret (intent of) the environment, decide on and plan their own behavior, and act safely based on their interpretations and subsequent decisions.

The “SENSE” environment perception function combines different sensors such as cameras, radars, lidars, ultrasound sensors that produce data that are integrated into a single sensor fusion model. Combining different sensor technologies compensates for individual weaknesses under the various environmental conditions and is the only way to come to a robust “SENSE” function. The sense functions evolve toward distributed model-based or AI-inference processing at the sense-node and in the central cognition, supported by edge-intelligence for training, and for real-time environment models from the Internet of Vehicles (IoVs). The concepts presented in this paper extend the findings and the experience of the authors working with electric, autonomous/automated, and connected vehicles technologies and applications, combined with the research and deployment of IoT/IIoT technologies across various industrial sectors ^1^^,^^2^^,^^3^.

The “LOCATE” function is using high definition dynamic maps, a common representation and encoding for 3D map data (IEEE P2751, 2017), GPS/GNSS information, local correction data to GNSS, RT2X, network positioning to create a semantic understanding of the context and the relative position in the operating environment of the robotic things.

The “CONNECT” function provides the communication means between robotic things and everything around (RT2X), while the “LEARN” function addresses the activities related to training and learning of the robotic things, as result of the experiences in various use cases and scenarios.

The “THINK” and “ACT” functions combine the interpretation of (intentions of) the environment with the goal of the journey, to follow a route, and path and detailed actions toward the robotic things' actuators. It involves deterministic calculations assessing uncertainty and inaccuracies to minimize the risk of accident and determine the most optimal route.

The “COLLABORATE” function is addressing the activities with other robotic things, autonomous vehicles, animals, environment, infrastructure (physical and digital, edge/cloud, etc.), and humans within a shared space, or in close proximity to produce, create, achieve shared goals, and minimize the risk of accidents and dangerous situations. The collaborate function incorporate one or several features such as natural or non-natural language communication, tactile interaction, safety-rated stop monitoring, teaching by demonstrations/examples, speed and separation monitoring, and power/force limiting, etc.

The IoRT is built upon the collaboration of intelligent robotic things to overcome challenges related to the system reference architecture, design, development, deployment, integrated devices and platforms management, business models and human involvement. It also must consider the integration of legacy systems and other IoT/IIoT applications.

These requirements are challenging for robotic things' computing, control, cognition, and connectivity platforms. They involve the introduction of service-oriented communication and dynamic operating systems, virtualizing functions, and distributing the functions between the IoRT platforms, the edge and cloud functions to meet the requirements for real-time, functional safety and security. Using dynamic control units is allowing to add functions (e.g., updatability, upgradability, learning, etc.) that are not available when the robotic things are operating isolated. A survey of safety-critical advanced robots is given in Guiochet et al. (2017) by analyzing the main issues, research work and challenges in the field of safety-critical robots, linking up dependability and robotics concepts.

In this context, determining the right combination of centralized and decentralized information processing is essential to realizing the optimal design and functionality of IoRT applications.

## Enabling Technologies

The combination of robots, AI [e.g., Artificial Neural Network (ANN), ML, DL, fuzzy logic, Particle Swarm Optimization (PSO), or other AI methods and techniques] and IoT/IIoT increase the IoRT capabilities to complete compound and multiple activities autonomously. Integrated into IoRT applications, robotic things can exchange information, collaborate with each other and with humans, facilitating high-quality information/knowledge exchange among them and with humans. By using ANN (Razafimandimby et al., 2016, 2018), the authors addressed one of the critical technologies for maintaining the connectivity between IoRT devices and provide the desired Quality of Service (QoS).

The IoRT has its technological basis in the convergence of multiple technologies: IoT/IIoT, autonomous robotic systems, intelligent connectivity, distributed and federated edge/cloud computing, AI, DTs, DLTs, VR/AR, and swarm technologies. It provides a technological basis for the development of next-generation IoT/IIoT technologies and the integration of these technologies with autonomous systems.

The sections below highlight and briefly describe several enabling IoRT technologies that are needed for the future development of IoRT applications and services.

### Internet of Things and Industrial Internet of Things

The IoRT is based on technologies derived from the IoT/IIoT paradigms that were developed during the last years. The IoT/IIoT technologies and applications are described in several published survey papers that cover different aspects of the IoT technologies and applications (Al-Fuqaha et al., 2015; Vermesan and Friess, 2015, 2016; Vermesan and Bacquet, 2017, 2018; Colaković and Hadzialic, 2018; Perrone et al., 2019; Sabry et al., 2019; Ud Din et al., 2019). The new enabling technologies for IoRT extend the existing IoT/IIoT and bring new requirements for the emerging IoT/IIoT technologies as emphasized in the next subsections.

The combination of AI and IoT/IIoT, the artificial intelligence of things (AIoT) is enabling and accelerating the developments of IoRT applications to achieve more efficient IoRT operations, improve humans-machines interactions and enhance data management and analytics. AI is used at the robotic functions and at IoT level to transform data into useful information for improved decision-making processes, thus creating a foundation for new services and intelligent collaboration among IoRT devices, fleets, and applications. AI adds value through machine learning capabilities, and IoT adds value to AI through connectivity, signaling, and data exchange.

The TIoT/TIIoT has started a conceptual shift from content-oriented media to sense/act/control-based media by implementing the real-time communication of haptic information (i.e., sensing/touch/feel, actuation, motion, vibration or surface, pattern/form/consistency) over the Internet, and the technology is part of the next evolution of the tactile Internet (TI). The principles apply to autonomous/automated and remote driving, virtually-coupled train systems, robotic devices, such as Unmanned Aerial Vehicles (UAVs), and other terrestrial, maritime and aerial autonomous, intelligent and cooperative mobility systems that have severe constraints in terms of latency, robustness, reliability, availability, and stability control (Sharma et al., 2019).

Internet of Things Senses (IoTS) as an extension of the TIoT/TIIoT concept involves technology interacting with our senses of sight, hearing, taste, smell, touch, enabled by AI, VR/AR, intelligent connectivity, and automation. The IoTS developments are key for the IoRT considering that the cognitive decision-making capabilities of the devices can be implemented by machine learning algorithms implemented into the robotic thing or at the edge, with the IoRT devices' memory represented by the data collected, the maps/environmental models, the eyes of the IoRT devices implemented by different perception sensors (e.g., cameras, radars, LiDARs, ultrasound, etc.), the ears represented by various microphones and IoRT to X communication, while the reflexes/coordination and the movements are implemented by the propulsion functions and actuators control.

The remote transmission via the Internet of the senses and the combination of the information from GPS sensors, accelerometers to measure motion, which way is up, gyroscopes to determine a twist and identify the orientation of the IoRT devices, ultrasonic sensors to detect objects in proximity, support operating the IoRT devices and creating the content and context for IoRT applications. The fusion of senses can support the IoRT functions and the simulation of different scenarios using virtual reality to replicate the physical movements in the real world and using ultrasonic technology to continuously provide distance measurement in three-dimensional space.

The tactile IoT/IIoT enables the real-time remote control and physical (haptic) experiences, and TIoT/TIIoT capabilities support the creation of a spatial safety zone that can interact with other nearby objects connected to robotic things that are part of IoRT applications. The safety zone concept applied to mobile robotic devices in IoRT applications allows the protection of other robotic devices, humans, animals, objects co-existing and operating in the same spatial environment. Robotic things have the capability to detect safety-critical situations, analyse the situations and decide how to react in real-time to avoid injuries, accidents, and warn other objects, robotic devices, and humans of imminent hazardous/risky/threatening situations. In production environments, occupational safety improves as production machines or robotic devices (static or mobile) can detect and avoid injuring people or colliding with other robotic devices in their proximity or surrounding area.

TIoT/TIIoT is believed to make it possible to create “avatar” collectives spanning different application domains and, therefore, cover heterogeneous robotic platforms (Haddadin et al., 2019). The developments of tactile robots enable the seamless interaction with heterogeneous systems like industrial assembly lines, service robots, automated medical units, employing robotic technology (IEEE P2730, 2019) deep sea, and space exploration units with further research focusing on robotics, multimodal teleoperation, wearable technology, distributed computing, or network technology.

### Autonomous Robotic Systems

Autonomous robotic systems are an essential enabling technology for developing the capabilities of individual IoRT devices, integrate them into platforms and allowing the creation of collaborative fleets of heterogenous IoRT devices for various applications. Autonomous robotic systems are expected to operate more seamlessly within the humans' environments and to achieve this they must integrate technologies providing senses (e.g., sight, hearing, taste, smell, touch) that human beings use. In this context, the IoRT devices can better operate with humans (e.g., humans-machines interfaces), and humans can better interact with the digital, virtual, and cyber worlds.

Enabling technologies for IoRT include both symbolic and sensory-based robot control and learning in the context of autonomous systems. The biological inspirations, including the social characteristics of insects and animals as part of the design of multi-robot systems, are key for IoRT developments in order to use local control rules of various biological societies (e.g., ants, bees, and birds) to the development of similar behaviors in cooperative IoRT systems.

The autonomous robotic systems technologies relevant to IoRT refers to architectures, localization/mapping/exploration, object transport and manipulation, motion coordination, reconfigurable robots, and distributed learning. The autonomous robotic systems technologies used in IoRT applications include reconfigurable robotic systems, multi-robotic things motion planning, traffic control and movement in IoRT formations and architectures for multi-robot cooperation.

Autonomous robotic systems as part of TIoT/TIIoT and IoTS require the integration of technologies that mimic the feeling of embodiment on passive touch, touched by oneself (self-touch) or by another human or robotic thing (e.g., affective social/interpersonal touch), to provide interfaces that approximate the capabilities of human skin (Beckerle et al., 2018). Tactile feedback technologies are key for assistive robotic devices used in various IoRT applications.

### Intelligent Connectivity

Connectivity for IoRT is largely focusing on wireless communication technologies. The term intelligent connectivity is used considering the interactions and combinations of wireless technologies (e.g., cellular 5G and beyond, or other wireless technologies), IoT/IIoT and AI techniques and methods.

Developing robotic things with robust and resilient wireless/cellular communication is key for IoRT applications. The communication channels used by IoRT applications could be wired or wireless, cellular, optical, sound, voice, images/videos. The communication networks can be based on any of a variety of protocols, such as TCP, UDP, 802.15.1, 802.15.4, 802.11 or 4G/LTE/5G, and beyond.

For mission and safety-critical IoRT applications where latency, reliability, and throughput are key requirements, centralized processing is substituted with edge-distributed processing, including analytics at the edge based on AI techniques and methods. Edge-distributed processing uses multi-access edge and fog computing technologies, and intelligent connectivity provided by wireless and cellular communication (4G/5G and beyond). This concept allows IoRT applications to process information locally and apply AI algorithms to the collected data using local learning, which scales down the demand to transfer large amounts of information to the cloud, store data locally, and reduce the overload of the connectivity links.

Intelligent connectivity networks can facilitate energy-efficient and high-performance information transfer and processing. Furthermore, edge network intelligent infrastructure can be implemented using Neural Networks (NNs), ML, and other AI techniques, to provide decentralized data analytics, automated network management, and the sharing of contexts and knowledge for IoRT or other IoT/IIoT applications. The cognitive capabilities of IoRT devices, combined with the cognitive capabilities embedded in connectivity networks, can perform functions embedded within the network infrastructure to supplement IoRT platforms' capabilities. In this scenario, knowledge generated by the intelligent connectivity network and by robotic devices can be used by the network itself, as well as in IoRT or other applications outside of the network.

### Distributed and Federated Edge/Cloud Computing

**Figure 7:**
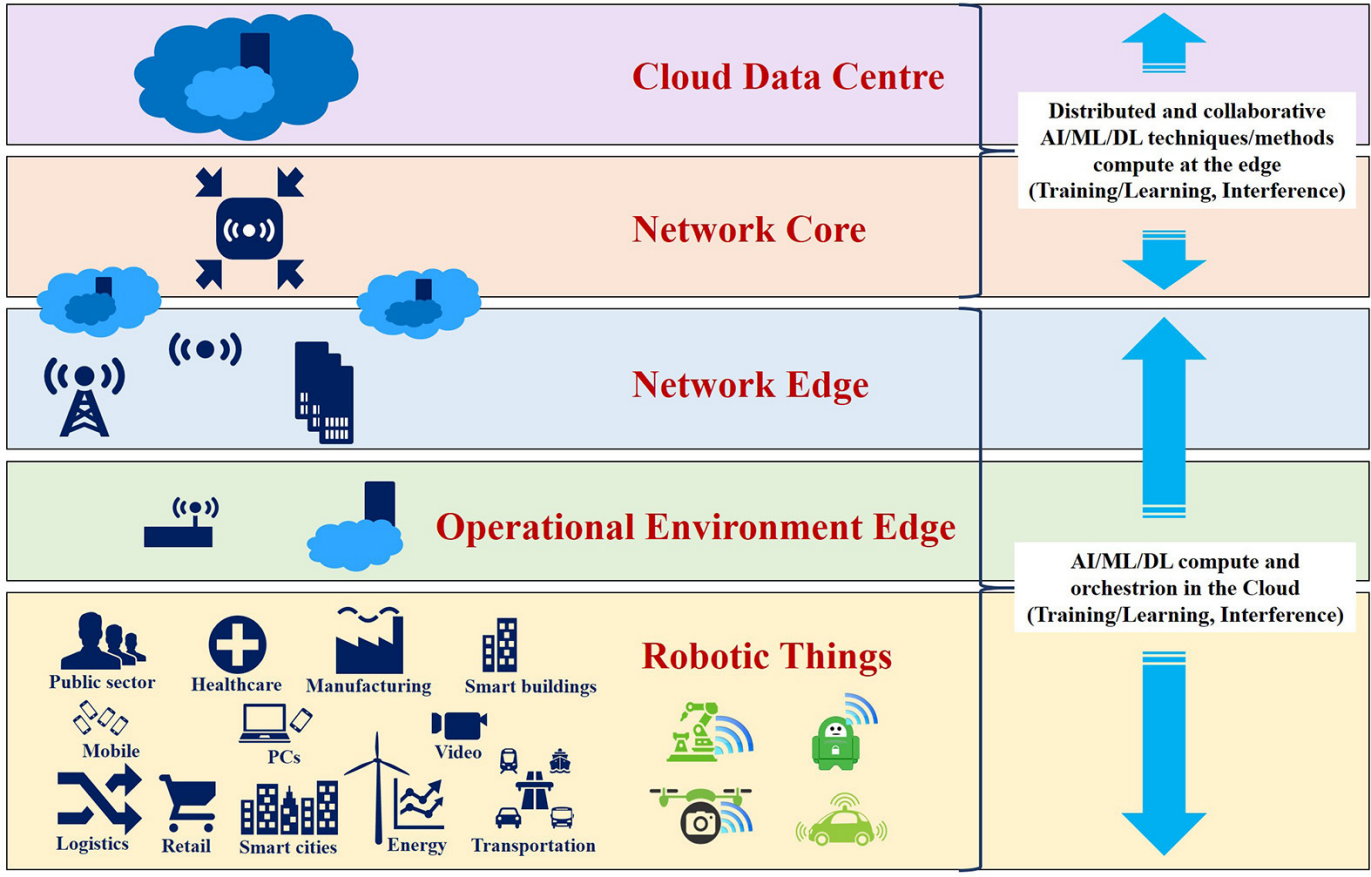
Distributed and collaborative AI approach across device-network edge-cloud layers.

The edge computing technology is suitable to deal with the complexity of IoRT technologies using distributed AI models at the edge for offloading Deep Learning (DL) computation (Li et al., 2018; Nikouei et al., 2018; Ren et al., 2018; Han et al., 2019) from end robotic things devices to edge and cloud as illustrated in Figure 7.

Edge computing is enhancing the IoRT, accelerating the development of high-performance IoRT devices using AI/ML techniques and embedded security for addressing edge processing. Edge computing provides mechanisms for distributing data and computing at the edge, which makes IoRT applications much more resilient to malicious and no malicious events. Distributed deployment models are expected to address more efficient connectivity and latency challenges, bandwidth constraints and higher processing power and storage embedded at the edge of the network. Using the edge computing layer of the IoRT architecture efficiently, it is possible to move most of the data traffic and processing closest to the end-user applications and devices that generate and consume data. The use of IoRT edge capabilities and diverse edge systems, centralized cloud services will enhance the functionalities of cloud technology to provide, manage and update software and services on edge IoRT devices. Centralized cloud services could become hubs in coordinating and federating operations across highly distributed edge robotic things and in aggregating and archiving data from the edge or intermediate gateways and servers. The centralized cloud services for intelligent IoRT applications will be used as robust and additional scalable machine learning and sophisticated processing capabilities linked to traditional back-office processing.

### Artificial Intelligence

The field of AI is pivotal for the developments of IoRT. The AI algorithms are enhancing the capabilities of the specific robotic things. The AI technologies are applied to optimize the sensor fusion capabilities of the IoRT devices (e.g., cameras for sensing the sight, chemical sensors for identifying the smell and taste, and microphones for hearing, pressure sensors to detect touch/pressure) and extract patterns from data to improve the cognition and decision-making processes. AI techniques and methods are implemented in the different layers of the IoRT platforms to provide analytics and insights and optimize the functions of the individual robotic things, and their collaborative behaviors as a fleet.

Convolutional Neural Networks (CNNs), is a type of Deep Neural Networks (DNNs), used to analyse and extract visual features from images. The techniques are designed for partitioning, and de-noising monitored signals to increase the performance of the recognition function by achieving high detection rates of quality variations or potential faults (Liang et al., 2019). The move from central computation to edge/fog nodes, allows IoRT applications to deal with extremely large data sets. This approach is also used in the case of efficient manufacturing inspection systems by combining AI techniques with processing at the edge by adapting a CNNs model to the fog computing environment that significantly improves its computing efficiency for an inspection model, that can indicate the defect type and its degree (Li et al., 2018).

At the robotic thing level, perception devices, such as cameras, are used for video capture, video data compression, video image pre-processing, and segmentation. Collaboratively training a context and scenery-aware adaptation model with the information received from different video capture devices allows for better object recognition accuracy. In order to balance the offload of the DL computation to IoRT devices, edge servers or cloud, an optimal offloading strategy must be determined. This needs to consider the trade-offs among critical metrics such as network condition, video compression, data rates/usage, power consumption, processing delay, frame rate, and processing accuracy of analytics. At the edge level, many distributed edge robotic thing devices can cooperate in providing better services. Edge computing federation and distribution of functions for compressing the DL model at the edge layer can improve the overall performance of the system. The cloud can assure the integration of DL models between the edge computing layer and update the parameters of the distributed DL models on edge devices (Ren et al., 2018). In the case where the edge infrastructure is unable to provide a reliable service with required quality (e.g., detecting objects/patterns with low confidence), the computing power and global knowledge in the cloud infrastructure can be used for processing /assisting the edge nodes in updating DL models.

### Digital Twins

The digital twin (DT) approach can be applied to IoRT devices and applications as a virtual representation of a robotic device across its lifecycle using real-time information received from physical IoRT devices to construct an optimal digital model, which can be used to simulate the physical IoRT device and, along with other devices, offer insights into application scenarios, optimization, performance and potential issues in real-time. As IoRT cognitive capabilities are based on AI techniques and algorithms, learning, and reasoning processes must be connected to the digital twin. In this context, the digital twin evolves and increase its capabilities, acting like a real, physical robotic device (Hoebert et al., 2019) and an interface for human-robot interaction (Pairet et al., 2019). Virtual simulations using the IoRT digital twins can support detecting new issues, testing new settings, identifying, and comparing different use cases, analyzing different operational and behavioral solutions, and creating various scenarios in a virtual, digital, or cyber environment.

Considering that the operations implemented by the digital twin could also be performed by the physical robotic device in specific conditions, twins can be designed to provide feedback as the IoRT application is refined, or an IoRT device twin can be used as an advanced “avatar” prototype of itself before the physical “thing” is built.

Developments in AI, IoT, and connectivity technology (Alsamhi et al., 2019b) are enabling the IoRT applications to improve energy efficiency and reduce power consumption, thereby leading to lower costs and lower latency. All these support faster decision making and seamless connectivity leading to more accurate insights. The robotic things digital twins support the simulation of more optimal “mission” scenarios and improved dependability (e.g., security, safety, availability, connectability, resilience, reliability, maintainability, privacy) which in turn leads to more trust.

### Distributed Ledger Technologies

A distributed ledger is a database distributed across various locations (or network nodes) and any change to the databases requires authentication by multiple instances (e.g., majority consensus) that increase the security test. This is creating shared accountability in the network, and the authority is shared between the different network actors (nodes).

DLTs are intertwined with IoT platforms and used to provide efficient data management in terms of security, privacy, and safety (Papageorgiou et al., 2020).

The blockchain refers to a DLTs solution where data from different transactions is linked, hashed, and organized per unit, one block at the time and each block is cryptographically “sealed.” The unique seal is the start of the next block of transactions that creates the blockchain structure. Examples of DLTs that are classified as blockchains' applications are Bitcoin, Ethereum, Neo, Stellar, Hyperledger, etc. In this context, the blockchain is the mechanism that allows the implementations to work, and the implementations are applications that use blockchain. The main characteristics of blockchains are the decentralized architecture, “trustless” system properties, the existence of consensus mechanisms, the maintenance of the history of transactions and the insurance of immutability.

The integration of hyperconnectivity, IoT/IIoT, AI, DLTs blockchains (Lopes and Alexandre, 2019; Lopes et al., 2019) and edge computing requires the next generation IoRT technologies to address these challenges. The description of the proper business models (Vermesan et al., 2016) and governance frameworks to support data/information flow across IoRT autonomous systems, the identification of liability in case of any issues and the understanding of the means to overcome the technical fragmentation in the IoRT are critical for the adoption of IoRT applications.

The blockchain (Ferrer et al., 2018) integrated into IoRT allows AI-based edge and cloud intelligence solutions for robotic things to be securely upgraded and enhanced through training, machine to machine learning and updated in real-time with new and improved skills.

### Virtual and Augmented Reality

Increased cognitive capabilities at the edge of IoRT applications allows the integration of immersive technologies (i.e., VR and AR) into human-robotic device interfaces, as well as interactions between robotic devices and the interfaces of IoRT platform systems. Cognitive capabilities based on new AI algorithms have prompted the need to increase the trustworthiness of IoRT systems by strengthening end-to-end security, electronic identities, services, and portable data/knowledge security. The transition from centralized to future distributed IoRT architectures requires concepts that address scalability, end-to-end dependability, privacy, and intelligent connectivity. The use of edge computing and software virtualization of functions and rule-based policy implementation requires a good flow of data and sharing of information and knowledge between IoRT applications/services running at the edge or in the cloud while ensuring the integrity and privacy of data.

VR/AR can be used in IoRT applications for learning, navigation and support functions (Vermesan et al., 2017b). While VR simulates environments, AR superimposes computer-generated information onto the real world, ensuring spatial and temporal synchronization between the digital information and physical world and enabling real-time interaction (Craig, 2013; Vermesan et al., 2017b).

AR tools allow IoRT designers to build up and create complex planning scenarios for robotic things in real-time by using the “digital twins” of robotic things. Unlike VR environments, this eliminates the need to model the dynamics of both the robotic thing and the physical environment. The AR framework builds a model of the physical world that serves as the reference for training and validating algorithms related to perception, motion planning, and control. For example, AR can be used to evaluate a robotic thing's capability to plan a safe path to a target location in a real outdoor scenario while the planning scenery is augmented dynamically by virtual objects (Gianni et al., 2014; Vermesan et al., 2017b).

The integration of the capabilities offered by AR, VR, DT, AI, systems to visualize virtual 3D models of the real world evolving into smart and interactive environments related to the context of things for physical objects of IoRT augmentation. The IoRT augmentation comprises of methods, techniques and technologies that are applied to improve the sensing, action, or cognitive abilities of IoRT devices. The concept expands to humans by providing an interactive digital extension of human capabilities (e.g., replication, supplementation) by using IoRT sensing and actuation technologies, AI, fusion and fission of information, AR, VR, and digital twins (Kuts et al., 2019a) to improve human productivity and capabilities.

### Swarm Technologies

The swarm technologies and swarm robotics (Dorigo et al., 2014) are focusing on the study of how intelligent systems comprising of multiple autonomous robots are used to perform collective tasks. Swarm robotics (Tan and Zheng, 2013) technologies are merging with the IoRT developments featuring self-organizing characteristics for multi-robot systems with high redundancy and requiring scalability, flexibility, and robustness.

The swarm technologies address algorithms to flock, disperse, aggregate, forage, and follow trails by applying the dynamics of ecosystems found in nature for the development of multi-robot teams that demonstrate emergent cooperation as a result of acting on predefined interests and goals.

The swarm technologies are used for IoRT applications that exchange information and create collaborative networks among various fleets of IoRT devices, with the fleets of robotic swarms characterized by their robustness to failure and scalability, due to the simple and distributed nature of their coordination (Ferrer, 2016).

### Platforms Technologies

The IoRT platforms main functions are to facilitate communication, data flow, device management for IoRT devices and enable the collaboration between IoRT devices within and across different platforms with the aim to build various IoRT applications using the IoRT platforms frameworks.

IoRT platforms technologies need to provide flexibility (capability to deploy IoRT devices in different contexts), usability (capability to make the user experience easy including humans-machines interactions) and productivity (enabling service creation to improve efficiency, but also enabling new service developments).

The IoRT platforms enable IoRT applications to connect robotic things, devices, applications, and people to data at the edge, cloud, and control centers.

The IoRT platforms architectures allow robotic things, locally embedded and/or distributed intelligence, and smart networks to interact and exhibit smart behavior and ultimately create open and sustainable marketplaces for large-scale complex and heterogeneous IoT applications and services. In this respect, IoRT platforms technologies need to support heterogeneous IoRT devices, address data ownership and the implications for security and privacy, provide data processing and data sharing capabilities (Mineraud et al., 2016), offer various tools and SDKs to application developers, assure the completeness of an IoT ecosystem, and the availability of specific IoRT marketplaces.

An overview of different IoRT enabling technologies with references to the relevant work and contributions is given in Table 1.

**Table 1:** Overview of different works involving approaches for IoRT enabling technologies.

**Enabling technology**	**Work**	**Topic**	**Findings**
Internet of Things and Industrial Internet of Things	Vermesan and Bacquet, 2018	Next-generation IoT distributed intelligence at the edge and human machine-to-machine cooperation.	IoT/IIoT continues to evolve with new technologies and applications, embedding ubiquitous hyperconnectivity (5G and beyond), edge computing, distributed ledger technologies (DLTs) and artificial intelligence (AI). Next-generation Tactile IoT/IIoT builds a real-time interactive system between the human and the machine and introduces a new evolution in human-machine (H2M) communication. Tactile IoT/IIoT enables the transfer of physical “senses” (e.g., sense/touch, actuation, hepatic actions, etc.) in real-time form remotely and introduces a new paradigm shift to the skill-based/knowledge-based networks instead of content-based networks.
	Colaković and Hadzialic, 2018	Internet of Things review of technologies, challenges, and research issues	There is a need for a modeling methodology to select the corresponding model of IoT and computing systems integration. There is a lack of mathematical formulation and evaluation methods which include multiple metrics. The authors identified the necessity to propose a comprehensive IoT model which include all possible architectures, technologies, and integration possibilities. In this context, the requirement for a quantitative method of evaluating IoT system performances has to be used for selecting the corresponding integration model and technologies as well as for creating performance-based profiles of IoT applications.
	Ud Din et al., 2019	Internet of Things technologies and challenges	Investigated the IoT enabled technologies in terms of smart cities, heterogeneous IoT, fog computing, data mining, WSN-based data-centric IoT, cellular communication, context-awareness, virtualization, and real-time analytics.
	Sharma et al., 2019	Tactile Internet	View on wireless Tactile Internet (TI) along with a thorough review of the existing state-of-the-art, to identify and analyse the involved technical issues, to highlight potential solutions and to propose future research directions. Main technical requirements in this regard include ultra-low latency, ultra-high reliability, very high data-rate, energy efficiency, spectral efficiency, and network throughput. Three main paradigms of TI, are identified: haptic communications, wireless AR/VR and autonomous, intelligent and cooperative mobility systems.
	Haddadin et al., 2019	Tactile robots and Tactile Internet	The combination of rich tactile feedback with state-of-the-art robotics, technology, and algorithms is providing the potential of immersive connection to human operators via smart wearables and virtual reality/augmented reality devices, effectively creating real-world avatars. The paper address the ways to seamlessly interact with heterogeneous systems such as industrial assembly lines, service robots, automated medical units and evaluates the potentials and enabling technologies together with foreseeable application domains in the framework of the Tactile Internet.
	Afanasyev et al., 2019a	Internet of Robotic Things architecture and components	The paper provides an overview, an analysis and presents the challenges of possible solutions for the IoRT, discussing the issues of the IoRT architecture, the integration of smart spaces and robotic applications. The authors describe the integration of robotics technologies in IoT scenarios. Future research topics related to IoRT are identified as the requirements engineering and formal processes, modeling, security, and process reconfiguration techniques applied to multi-robot systems.
Autonomous robotic systems	Seminara et al., 2019	Active haptic perception in robots	The paper provides a reasoned, principled perspective on the connections between different taxonomies used in the robotics and human haptic. New-generation robots are increasingly equipped with more sensing components, and consequently, they are (to some extent) able to deal with highly complex and dynamic real-world tasks. Research in robotics can contribute to the design of novel human-like robotic hands, considering the transition from task-based to structure-based design. The advancements are both cognitive and physical in nature, based on efficient data representation, real-time processing, and embedded networking.
	Liu, 2019	IoT and robotics in intelligent manufacturing	The author examines how the merger of robotic and IoT technologies can advance intelligent manufacturing, thus enabling the creation of new, potentially disruptive services. IoRT shall advance beyond the terms of “Robot-enhanced IoT” or “IoT-aided robot”. Advance the ecosystems of cloud, IoT agents and robots that integrates both to promote the development of intelligent manufacturing.
	Batth et al., 2018	Intelligent robotics, concept, architecture, applications and technologies	The authors highlight a concept of architecture, which plays a significant role in the design of multi-role robotic systems for IoRT. The paper presents technologies behind IoRT, applications of IoRT and existing robotic systems based on humanoid, mobile, flying, and swarm envisaged for future IoRT systems.
	Alsamhi et al., 2019a	The convergence of Machine Learning (ML) and robotics communication	The paper addresses the convergence of ML and communication for collaborative assemblies of robots operating in the space, on the ground and in underwater environments. Improvements in swarm robotics applications are proposed based on addressing the issues like preventing collisions, keeping connectivity between robots, maintaining the communication quality, and ensuring collaboration between robots.
	Beckerle et al., 2018	Touch for embodiment in assistive robotics	The paper addresses the integration of objects into one's bodily self-representation, as a key aspect of human self-consciousness and cognition that can be extended toward robots is argued as being crucial for assistive technologies aiming at restoring, extending, or simulating sensorimotor functions. High-density and large surface sensing and stimulation are required to foster the embodiment of such assistive devices. Versatile and realistic tactile feedback covering the different facets of touch will enhance the usability and the user experience of assistive robots. Advanced robotic touch technology embedded into robotic artefacts will likely be the main tool to enforce a real synergy with users.
	Nikouei et al., 2018	Edge computing and implementation of Harr-Cascade and HOG feature extraction and SVM classifier, and a lightweightConvolutional Neural Network (L-CNN) for human detection	Performing computation near the source and destination, edge computing is promising to address the challenges in many delay-sensitive applications, like real-time human surveillance. Leveraging the ubiquitously connected cameras and smart mobile devices, it enables video analytics at the edge. Human object detection algorithms, namely Haar Cascaded object detector and HOG+SVM human detector, in the context of edge computing, are evaluated. The algorithms are implemented on an edge device, and the experimental results have verified that the proposed L-CNN algorithm has met the design goals.
Intelligent connectivity	Qi and Ma, 2018	Vehicular Edge Computing via Deep Reinforcement Learning	A knowledge-driven (KD) service offloading decision framework, which provides the optimal policy directly from the environment, is presented. The framework supports the pre-training at the edge computing node and is continually learning online when the vehicular service is executed so that it can adapt to the environment changes and learns the policies that are sensitive to experiences. The simulation results show that KD service offloading decision converges quickly, adapts to different conditions, and outperforms the greedy offloading decision algorithm.
	Razafimandimby et al., 2018	ANN, connectivity and IoRT	Intelligent techniques used to allow IoRT robots for provisioning desired QoS and reducing energy consumption are investigated. Motion control strategies which maintain global connectivity between IoRT robots to the desired QoS level using an IoT-based approach and a distributed trained ANN neural network controller are presented. Focus on capturing the trade-off between network coverage and communication quality expressed as RSSI level using the proposed algorithms allows the whole IoRT robot network converges to the desired distance, and hence the desired communication quality.
	Sharma et al., 2019	5G and beyond systems for achieving ultra-high latency (about or less than 1 ms) and ultra-high reliability	Potential enabling technologies across physical, Medium Access Control (MAC) and network layers are identified. Haptic communications demand for some specific requirements in terms of symmetric resource allocation in both the uplink and downlink, joint resource allocation in both the downlink and uplink, the consideration of bounded delay and guaranteeing the minimum rate throughout the haptic session. Challenges, (e.g., communicating kinesthetic information between the master and slave ends in a teleoperation system that requires a high packet transmission rate of about 1,000 or more haptic data packets per second), leading to an inefficient data communication due to the depletion of the network resources are identified for the effective design and operation of teleoperation systems.
	Tan and Hu, 2018	Communication and computing design in vehicular networks	Vehicular networks are based on advanced communication technologies and data collection techniques to improve safety, enhance efficiency, and decrease traffic congestions in mobility systems. The resource allocation policy is designed by considering the vehicle's mobility and the hard service deadline constraint. A deep reinforcement learning with the multi-time scale framework is used to address the vehicular networks operational requirements, and a mobility-aware reward estimation for the large timescale model is proposed to mitigate the complexity due to the large action space.
Distributed and Federated Edge/Cloud computing	Han et al., 2019	The convergence of edge computing and DeepLearning (DL)	Edge computing is gradually being combined with AI, benefiting each other in terms of the realization of edge intelligence and the intelligent edge. Edge intelligence is expected to push DL computations from the cloud to the edge as much as possible, thus enabling various distributed, low-latency and reliable, intelligent services. Considering the multiple constraints for networking, communication, computing power, and energy consumption, edge computing architectures are optimized to achieve the best performance of DL training and inference. As the computing power of the edge increases, edge intelligence becomes common, and intelligent edge plays an important supporting role to improve the performance of edge intelligence.
	Li et al., 2018	Fog computing and Deep Learning (DL) for industrial applications	A system design based on the concept of fog computing to offload the computation from the central server to the fog nodes to deal with extremely large data is presented. The use of a convolutional neural network model to the fog computing environment, to improve the computing efficiency and work out an inspection model, that can simultaneously indicate the defect type and its degree is described.
	Capra et al., 2019	Edge computing and IoT	An overview of the main techniques to design hardware platforms able to cope with IoT requirements, by exploiting the edge computing paradigm is presented. Hardware architectures of typical IoT devices are discussed, and low-power techniques are evaluated. The algorithms for computer vision, speech recognition require high computing power and, a substantial amount of energy. The future research should address the optimization of the AI algorithms to meet the hardware and energy constraints dictated by the IoT platforms. Moving AI at the edge and using edge computing will optimize the IoT applications.
	Gudi et al., 2019	Fog robotics	Robots are limited by their own capabilities and, utilize cloud robotics to enhance their dexterity. This requires the sharing of information (e.g., maps, images, computing and processing resources, etc.). Transferring large amounts of data increase the bandwidth and the network congestion at backhaul and fronthaul systems resulting in high latency. Fog robotics can act as a solution by solving the problems of cloud robotics. The experimental results show that fog robotics reduces latency significantly compared to cloud robotics.
	Jain and Doriya, 2019	Security and cloud robotics	The authors review various security aspects confining more on the security details related to cloud robotics with security flaws as being major concerns that can affect the cloud robotics. Cloud computing suffers from various type of vulnerabilities like network-based attacks, data storage based attacks, virtualization based attacks that are sensitive to operating system-level attacks. These elements affect robotics that can be exposed to attacks such as the denial of service and dictionary attacks, etc. Focus on security methods and techniques that can be applied to cloud robotics to make it more safe and secure.
	Lopes et al., 2019	Robots, AI, blockchain	The paper proposes an architecture that uses blockchain as a ledger and smart-contract technology for robotic control by using external parties, Oracles, to process data. The concept allows to register events in a secure way, use smart contracts to control robots and interface with external AI algorithms for image analysis. The proposed architecture is modular and can be used in multiple contexts such as in manufacturing, network control, robot control, and is easy to integrate, adapt, maintain, and extend to new domains. Integration of AI with blockchain has benefits for swarm robotics and robotic hardware by using the global information within robotic swarms in a secure and validated way and a faster way to change the behavior of the network, which will ultimately lead to higher productivity and easier maintenance.
	Khelifi et al., 2019	Deep Learning at the edge	Applicability of merging deep learning (DL) models (e.g., convolutional neural network (CNN), recurrent neural network (RNN), and reinforcement learning (RL), etc.), with IoT and information-centric networking for Internet architecture, combined with the edge computing concept is presented. A CNN model can be used in the IoT area to exploit data reliably from a complex environment, while RL and RNN are integrated into IoT, which can be used to take the multi-modality of data in real-time applications.
	Wan et al., 2020	Cognitive computing and wireless communications on the edge	Perception capabilities of mobile healthcare robots based on special sensors and AI techniques are described. Promising solutions may arise from several state-of-the-art deep learning algorithms, including Convolutional Neural Network (CNN), and Generative Adversarial Network (GAN). Open research issues include intelligent communications, ground-breaking biosensors, cutting-edge AI, and state-of-the-art deep learning algorithms.
Digital twins	Malik and Bilberg, 2019	Digital twins of human-robot collaboration in production	A digital twin framework to support the design, build and control of human-machine cooperation is presented. Computer simulations are used to develop a digital counterpart of a human-robot collaborative work environment for assembly work. The digital model is extended for real-time communication with the physical system for performance optimization on a system level using a cloud-based service with real-time performance metrics, optimization analytics and alerts for a robot, continuously updating the digital twin.
	Kousi et al., 2019	Digital twin for adaptation of robots' behavior in robotic assembly lines	The use of digital world modeling techniques in hybrid production systems for enabling system reconfiguration through a shared environment and process perception is described. The paper proposes a digital world model infrastructure with three main functionalities: (a) Virtual representation of the shop floor, combining multiple sensor data, and CAD models [e.g., the digital shop floor is rendered in the 3D environment exploiting the capabilities provided by Robot Operating System (ROS) framework], (b) Semantic representation of the world through the implementation of a unified data model for representing the geometrical and the workload state, (c) Dynamic update of the digital twin based on real-time sensor and resource data coming from the actual shop floor. The communication and integration layer among the physical and the virtual agents are realized on top of the ROS framework. Future research
			requires the integration of the DT with: (a) the physical robotic set up to validate its performance and (b) high-level decision-making mechanisms allowing the reconfiguration of the system at shop floor level through task re-allocation based on the real-time production needs (new product variants etc.).
	Kuts et al., 2019b	Digital twin control and simulation of the industrial robotic cell using virtual reality	The authors propose to create an Industrial Digital Twin (IDT) – as a digital copy of the real manufacturing system, which can be controlled and programmed in real-time directly from the computer application model of the industrial robot. The paper provides a precise model of the robot and the developing software package to control and program it directly from VR. Future research is needed to address the development of a modular approach and the optimization of synchronization framework between virtual and real-world, including the model optimization of the robot digital twin.
Distributed ledger technologies	Ferrer, 2016	Blockchain and robotic swarm systems	The combination of blockchain with distributed systems, such as robotic swarm systems, can provide the capabilities to make robotic swarm operations more secure, autonomous, and flexible. Security, decision making, behavior differentiation, and business models for swarm robotic systems are described by providing use case scenarios. Robots may be able to function in diverse and changing environments if their operation corresponds to different blockchain ledgers that use different parameters, without any change in their control algorithms.
	Lopes and Alexandre, 2019	Blockchain integration with robotics and AI	Different methods and platforms that leverage the power of blockchain into robotic systems to improve AI services, or to solve problems that are present in the major blockchains, which can lead to the ability to create robotic systems with increased capabilities and security are described. Blockchain can help to automate processes with the support of smart-contracts and enable systems to have improved security and more traceable processes. Blockchain introduces a way to trust the data, trust other participants, and to conduct internal and external changes by having certified information regarding the whole system.
	Afanasyev et al., 2019b	Blockchains for multi-agent robotic systems	The blockchain could play a significant role in multi-agent system applications. The analysis allowed the authors to identify groups of tasks for blockchain-based multi-agent robotic systems, which are proposed for classification. Future open issues include the development of a conceptual model of information support groups of robots during the task performance, work on a consensus protocol for a group interaction verification before launching a task based on the information from a distributed ledger, validation method for task performance and development of multi-agent system architecture.
	Gianni et al., 2014	Augmented reality environment and mobile robots	The authors introduce a development tool for constructing, in real-time, complex planning scenarios for robots, eliminating the need to model the dynamics of both the robot and the real environment as it would be required by whole simulation environments. The AR framework is used for evaluating the capability of the robot to plan safe paths to goal locations in real outdoor scenarios, while the planning scene dynamically changes, being augmented by virtual objects.
	Malik et al., 2020	Virtual reality in manufacturing and human-robot workspace	Technological development in virtual reality for the design of human-centered production systems requires a unified framework to integrate human-robot simulation with VR. The simulation as an event-driven simulation is used in estimating the human-robot cycle times, developing process-plans, layout optimization, and robot control programs. The simulation is utilized in VR to interact with the production equipment and particularly with the robots.
	Makhataeva and Varol, 2020	Augmented reality for robotics to enhance the perception	Four categories are analyzed: (1) Medical robotics: Robot-Assisted surgery (RAS), prosthetics, rehabilitation, and training systems; (2) Motion planning and control: trajectory generation, robot programming, simulation, and manipulation; (3) Human-robot interaction (HRI): teleoperation, collaborative interfaces, wearable robots, haptic interfaces, brain-computer interfaces (BCIs), and gaming; (4) Multi-agent systems: use of visual feedback to remotely control drones, robot swarms, and robots with a shared workspace.
	Tan and Zheng, 2013	Swarm robotics	Swarm robotic algorithms are presented, including cooperative control mechanisms in swarm robotics for flocking, navigating, and searching applications. Challenges are identified in how can the cooperative schemes inspired from the nature swarms integrate with the limited sensing and computing abilities for a desired swarm level behavior, in developing mathematical models to describe the swarm robotics system and predict the system behaviors at both individual and swarm level and in developing new architectures and strategies for integrating swarm robotics systems.
	Chamanbaz et al., 2017	Swarm technologies for multi-robot systems	The authors present the design of integrated hardware and software tools, enabling a wide range of multi-robot systems to collectively operate in a distributed manner. Future research directions include swarm algorithms in the software library, simulation techniques for collective behaviors prior to implementation onto the platforms. Swarm-enabling technology is seamlessly used with different mobile robots to facilitate studies of heterogeneous swarming.
	Schranz et al., 2020	Swarm robotics applications	The paper analyses swarm behaviors and categorize these behaviors into the spatial organization, navigation, decision making, and miscellaneous. The taxonomy is then applied to categorize several existing swarm robotic applications from research and industrial domains and give a comprehensive overview of research platforms that can be used for testing and evaluating swarm behavior, systems that are already on the market, and projects that target a specific market. Swarm behavior emerging from local interactions is hard to predict, and a proof of its eligibility for applications in an industrial context is difficult to provide. Existing communication architectures do not match requirements for swarm communication, which often leads to a system with centralized communication infrastructure. Testing swarms for real industrial applications is an issue since deployment in a production environment is typically too risky, and simulations of a target system may not be sufficiently accurate.
Platforms technologies	Bröring et al., 2018	IoT platforms interoperability	The authors describe the interoperability issues in the IoT platforms and ecosystems covering the interoperability aspects, challenges and approaches that cope with interoperability in the current existing IoT platforms and introduce insights regarding the future of interoperability by presenting possible solutions, and a possible IoT interoperability platform architecture. Layered approaches for interoperability allow the stakeholders or platform operators to select the best mechanism for interoperation. Management of such options provides coordination between layers, enhances cooperative solutions (e.g., gateways and network) and enables security management.
	Mahieu et al., 2019	Semantics-based platform robot interaction in IoRT	A platform approach based on a modular, data-driven workflow that allows developers of interacting services to determine the appropriate time, content and style of human-robot interaction tasks by reasoning on semantically enriched IoT sensor data is proposed in this paper. The platform abstracts the complexities of scheduling, planning and execution of these tasks, and can automatically adjust parameters to the personal profile and current context.
	Sabri et al., 2018	Semantic framework and IoRT	A semantic framework is proposed for context-aware IoRT systems to support the development of applications for monitoring and managing IoRT systems using a knowledge representation framework, called SmartRules, for context modeling. SmartRules as a production rules language enables reactive reasoning based on the closed world and unique name assumptions, allowing the generation of actions based on contextual information represented in a dedicated ontology language, called μ-Concept. An operational platform, centered on the notion of a manageable object (MO), is proposed to abstract the access to any physical or virtual device, which can communicate through the Internet. An integrated methodology and tools are proposed for guiding the development and deployment of context-aware semantic IoRT systems, and for defining context semantics and creating context management rules.

## IoRT Architectural Approach

IoRT is a relatively new field, and several attempts have been made (Ray, 2016; Batth et al., 2018; Yousif, 2018) to provide an IoRT architecture. These approaches are built on the IoT reference architectures developed many years ago and focusing only on one-dimensional layered architectural view.

**Figure 8:**
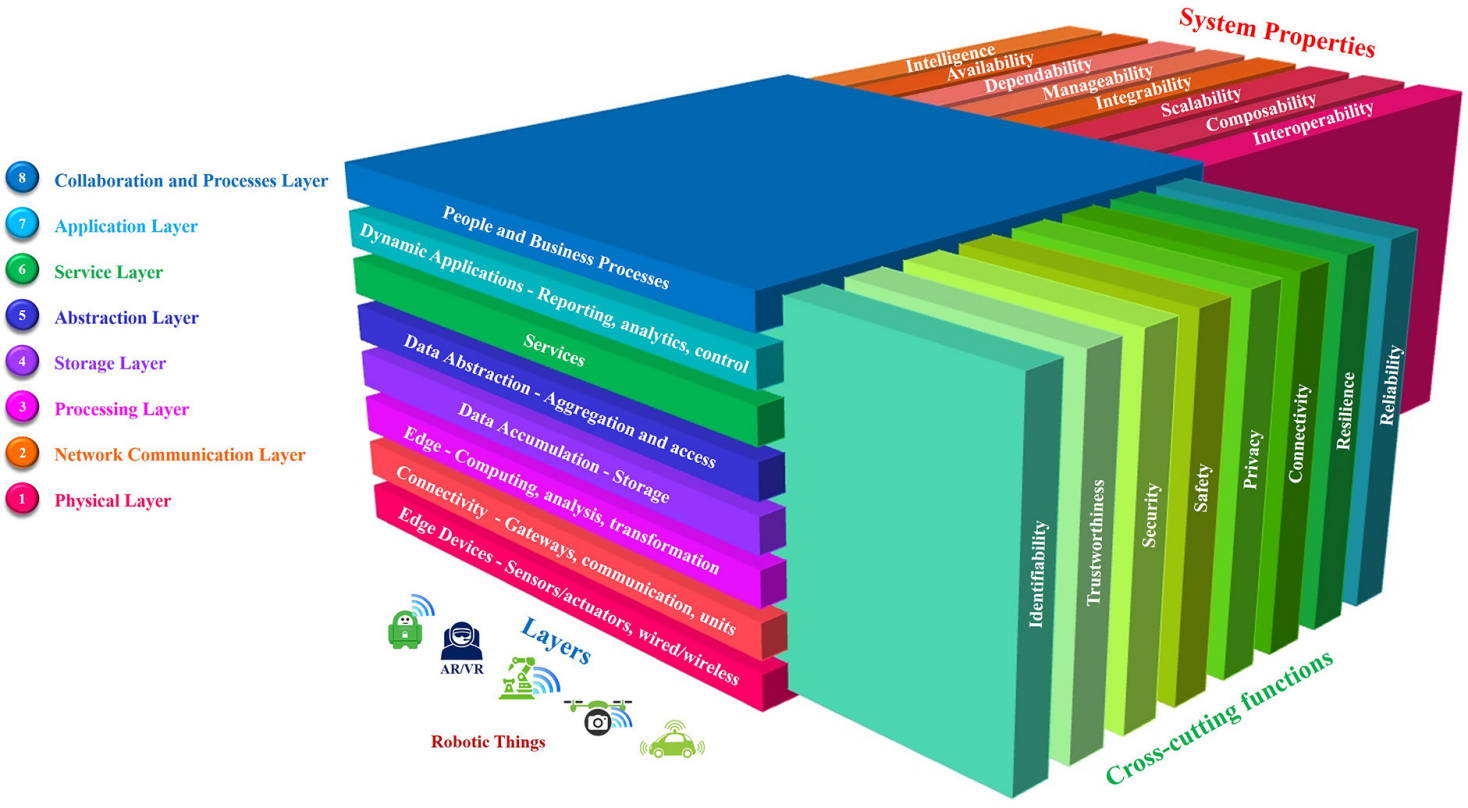
3D reference IoT/IIoT architecture applied to IoRT.

In this section, we present a 3D layered reference architecture of the IoRT, mapped onto the proposed 3D reference architecture for IoT/IIoT (Figure 8). The architecture is used as a source of information about the overall framework of IoRT technologies and applications that guides and constrains the instantiations of multiple solutions that are implemented in various IoRT use cases.

The architecture consists of a 3D representation of the key components of IoT/IIoT applications from the perspective of the eight domains layers, eight cross-functions and eight system properties. Different applications may require different components in the architecture depending on their requirements and specifications.

The 3D layered reference architecture of the IoRT supports the definitions in the early design phases of common data models, communication standards, exchange formats, common IoRT SW/HW building blocks and define reusable assets and models.

The IoRT reference architecture includes generic architecture patterns and layers, as well as the edge computing features and specific characteristics of IoT/IIoT architectures (as defined in IEEE P2413, 2019). The physical layer is represented by various sensors and actuators, IoT/IIoT, IoRT, and other intelligent autonomous devices. The IoRT devices are intelligent agents that can communicate and collaborate with each other and establish a multi-robotic system that offers new services through distributed actions. The network layer includes components that utilize a variety of protocols to communicate and control processes involving multiple robotic things. The layer can include routers, controllers and gateways to provide the required connectivity. The layered architecture patterns are mapped to the IoRT to provide an overall view of the different layers (physical to business) of IoT/IIoT systems, including two additional dimensions: cross-cutting functions and system properties.

Increasing the level of trustworthiness in IoRT systems requires addressing the end-to-end dependability of IoRT applications (e.g., safety, security, reliability, resilience, availability, connectability, and maintainability). In this context, the layered architecture presented (Vermesan et al., 2018) is used to capture the system properties and cross-cutting functions of a 3D IoRT-layered architecture (Figure 8). The architecture was proposed after investigating and analyzing different reference architectures concepts [e.g., Reference Architectural Model Industrie 4.0 (RAMI 4.0), Industrial Internet Reference Architecture (IIRA), Reference Architecture Model Edge Computing (RAMEC)], to understand the underlying elements, functions and definition, what they are used for, the goals, objectives, characteristics, key features, and properties. The goal was to determine common patterns for defining the architectural views, domains, functions, properties for the IoRT reference architecture. The key components of the existing reference architectures were compared with the definition of the functional and non-functional requirements and the specific characteristics defined by different IoRT use cases.

IoRT applications need to use new techniques provided by DLTs, swarm logic and AI to produce dynamically complex behavior, independently, or collectively, to respond and adapt to hacking, threats and cyber-attacks. Security by-design and distributed end-to-end solutions are needed to enhance the ability of IoRT technologies to deal with various cyber scenarios. The 3D layered reference architecture of the IoRT by introducing the system properties and cross-cutting functions views allows the implementations of IoRT applications using DLTs and AI methods and techniques at different layers of the architecture.

As IoRT applications' topologies address different requirements and industrial sectors (Jain and Doriya, 2019), security configurations and strategies have to be scalable and adapted to the specific context and environment where the IoRT devices are operating. Furthermore, performing routine over-the-air security updates of heterogeneous robotic devices that operate at the edge is key to keeping the whole application secure. IoRT dynamic device-orchestration techniques and security updates are essential as IoRT edge devices are vulnerable and can function as entry points for cyber-attacks, which are difficult to track, trace, isolate and, consequently they quickly spread throughout an entire IoRT or IoT/IIoT ecosystem.

The normal, safe, and secure functioning of IoRT applications relies on end-to-end protection at all IoRT architectural layers, as well as a platform's functions, from robotic devices to communications, edge, storage, services and applications. To be considered dependable, robotic devices must exhibit a high level of robustness/resilience against a wide range of various types of harmful attacks, including software, hardware, connectivity, and physical tampering, which enables the implementation/deployment of trustworthy IoRT technologies, solutions and applications. The 3D layered architecture allows integrating all these elements into the IoRT applications.

The 3D architecture is generic and offers a representation that can include different IoT/IIoT applications across different sector domains and is well-suited to IoRT technologies. The architecture includes the function-by-design concept with end-to-end functions addressed across the eight layers. This allows one to address various applications, including different IoT platforms and processing at the edge, fog and cloud, as well as device management, capabilities (including command/control of devices) and the inclusion of various gateways for implementation of different functions across the eight layers.

The 3D architecture also provides an optimized view of stream processing across the eight layers, enabling evaluation of the rules and functions for analyzing information streams.

By including elements such as intelligence, dependability, manageability, integrability, composability, and interoperability in the system properties view, they can be specified and implemented across the layers. For example, the intelligence system property can define machine learning components across layers for predictive algorithms to be executed on historical IoRT data, enabling predictive maintenance functions. Furthermore, information transformation across the eight layers and the aggregation of the IoT data stream can be specified for different applications, including protocol transformation, and interoperability interfaces.

### Moving From Centralized to Decentralized and Distributed IoRT Architectures

The mobile robotic things devices that are part of the IoRT have an operating time which is proportional to the activity type (e.g., static sensing, high-speed propulsion, heavy-duty manipulation, etc.), battery size/capacity, the number of tasks performed per activity cycle, data rate and volume of exchanged data/information with other robotic things, humans, and infrastructure.

The transfer of large data sets to a central cloud represents a very energy-consuming operation, and new computational paradigms are used and implemented for IoRT applications.

In future IoRT applications, the computation is not entirely performed in the cloud, and new orchestration methods are developed for distributing the power load among the nodes of the IoRT system, compress data, or transmit only the processed data (the so-called “smart data” to reduce the transmitted power requirements).

In this context, the 3D layered reference architecture of the IoRT divided into eight vertical layers can address the decentralized and distributed topology for future IoRT applications.

IoRT applications operate fleets of robotic things that are based on a set of cooperative rules that the individual IoRT devices have to follow, so they can perform the tasks in a distributed manner without centralized controls, which allows for scalable, flexible, and dynamic implementation architectures. This ensures that the autonomous functions of the IoRT devices are active, even if communication with the central unit for federating information is interrupted for a short time.

**Figure 9:**
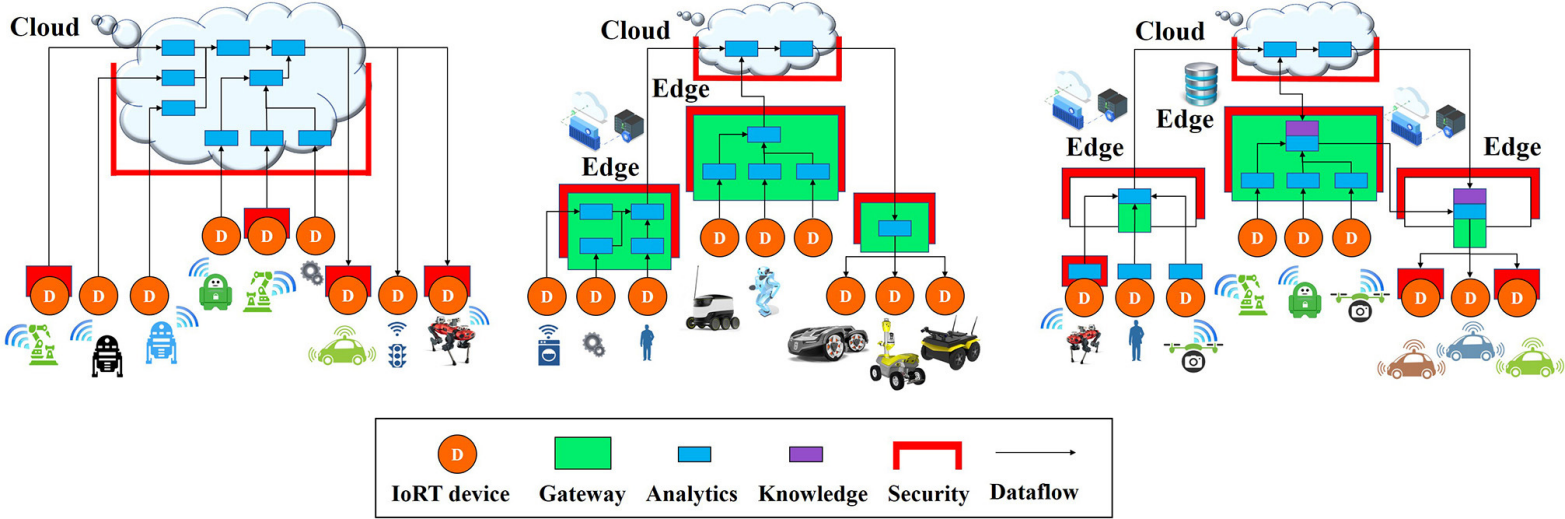
Transition from centralized to decentralized and distributed topology for IoRT applications.

The transition from centralized to decentralized topologies for IoRT applications is presented in Figure 9. In the edge computing framework, part of the processing is moved closer to data collection sources (e.g., robotic things, IoT/IIoT devices, etc.), and, only after first processing, collected data is sent to the cloud (Capra et al., 2019).

For a wider range of application scenarios, including Internet of Vehicles (IoVs) and IoRT, a wider range of intelligent services are still bounded due to several factors (Han et al., 2019):

- Cost: training and inference of Deep Learning (DL) models as part of AI techniques in the cloud requires devices to transfer large data sets to the cloud, thus using power and a large amount of network bandwidth.
- Latency: transmitting, processing the data, and using the services provided by the cloud applications cause significant transmission delays. For real-time, safety-critical scenarios, autonomous/automated vehicles cannot allow hundreds of millisecond delays that are caused by many cloud processing tasks (Khelifi et al., 2019).
- Reliability: wireless communications and backbone networks are the critical infrastructures on which cloud computing relies for providing reliable, robust, and resilient services. In the case of industrial manufacturing, IoRT, IoVs scenarios, intelligent services must be ultra-reliable and provide the required information and functions even when network connections are lost.
- Privacy: the information required for DL, ML, and other AI techniques involves a lot of private information. Privacy issues are critical to different sectors in which the IoRT devices operate and interact with other IoT/IIoT devices and humans.

In the context of IoVs, the move from a centralized to a decentralized solution is also applied to learning. A Knowledge-Driven (KD) service offloading decision framework for IoVs is proposed by Qi and Ma (2018) for providing the “optimal policy directly from the environment” by formulating the offloading decision as a long-term planning issue of the multi-task in service and exploring deep reinforcement learning to reach the optimal solution. The proposed framework “supports the pre-training at the edge computing node and continuous online learning when the IoVs' service is executed so that it can adapt to the environmental changes and learn the policy that applies” (Qi and Ma, 2018).

Moving from centralized to decentralized and distributed intelligent IoRT edge poses many challenges in the future. The open questions that still need solutions are how to optimally identify the services that are provided at the edge and in the cloud, how to enable in real-time the orchestration and exchange of information between the edge and the cloud, both for operations and learning, how to enable the intelligent collaborations and networking among the edge devices for implementing distributed architectures, and what computations should be embedded at the nodes at the edge and in the robotic things. In this context, the 3D layered IoRT reference architecture allows the partitioning of the functions of the IoRT system into different layers, platforms, cloud/edge providing a clear view where the different functional HW/SW, communication, processing, analytics components are implemented in the 3D stack.

## Intelligent Connectivity

IoRT applications rely on robust, resilient, and reliable connectivity networks, and the intelligent connectivity infrastructure needs to function as a continuum and interoperable network that can support heterogeneous devices with different intelligence capabilities and connectivity needs, depending on the application. Connectivity infrastructure needs to be flexible and adapt to the environmental context's requirements and planned/unplanned events and scenarios.

AI techniques, such as ML, are used as efficient tools to address the issues experienced in 5G wireless communications (e.g., caching, computing, and communication processes) for obtaining the operational requirements and “the cost efficiency of the vehicular networks” (Tan and Hu, 2018).

Wireless and cellular communication networks need to assure predictable/guaranteed latency to allow new connectivity, which has become the enabler for next-generation IoRT intelligent services. IoRT devices can use, for exchanging information and direct communication, peer-to-peer, and/or broadcast systems.

**Figure 10:**
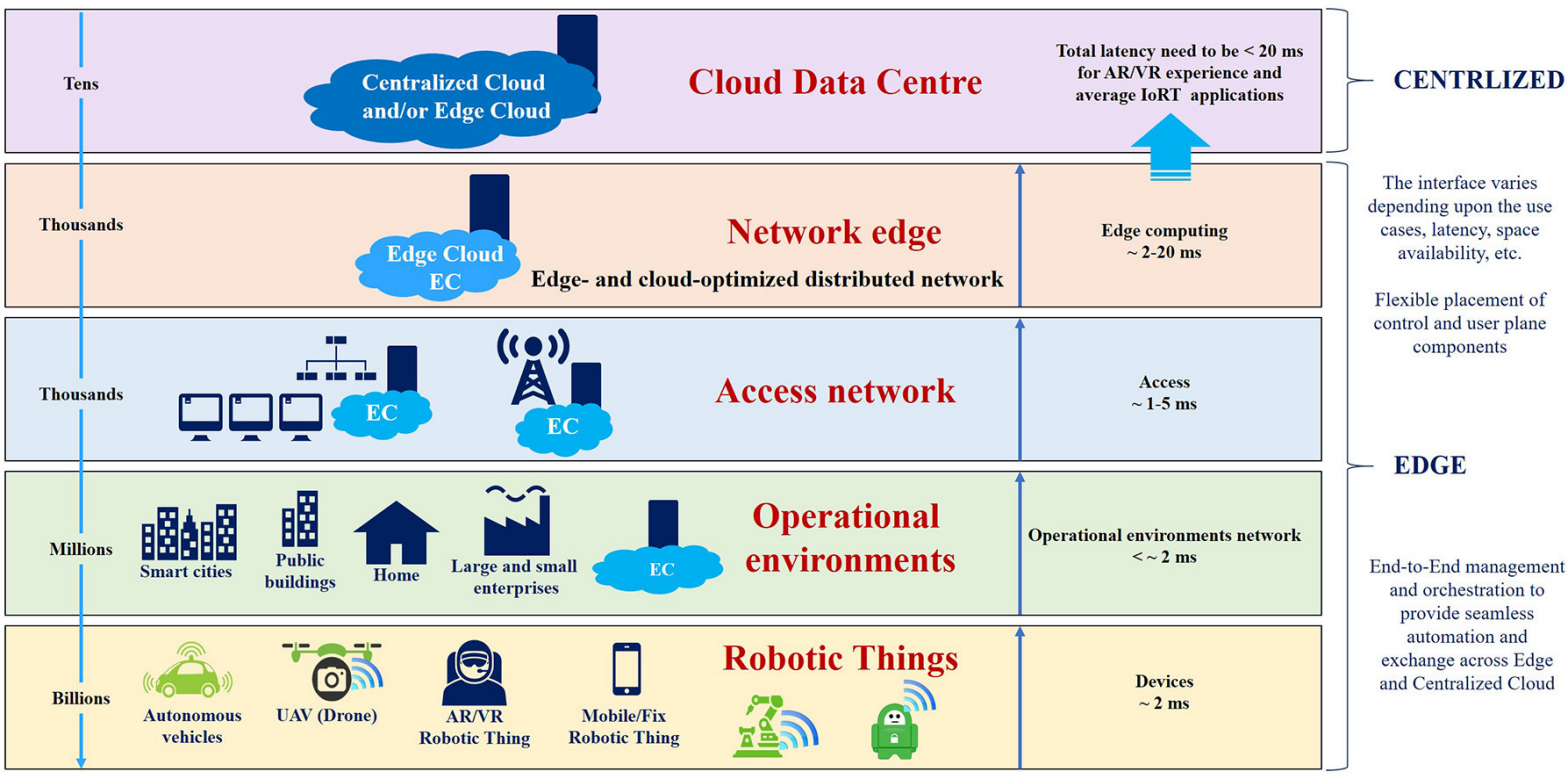
Edge and cloud optimized distributed network topology for IoRT connectivity.

Today's 4G cellular technology provides latency performance around 80–100 ms, while the new 5G technology will provide 1–10 ms latency because it uses new modulation schemes for network slicing capabilities, wireless access, automated network application lifecycle management, software-defined networking (SDN), and network function virtualization (NFV). Edge and cloud-optimized distributed network applications (Figure 10) are supported to address strict energy efficiency constraints requirements to cover large outdoor spaces, deep indoor/underground environments, or mobile things moving at high speeds. The figure illustrates the partitioning of different processing components for IoRT applications and the distribution of the functions between the edge and cloud. The illustration is mapping on the first four layers of the 3D layered IoRT reference architecture.

The requirements for mission and safety-critical IoRT applications are focused on latency, reliability and throughput, which are addressed by the 5G network's architecture and new functionalities. However, as 5G network implementation is based on software, risks related to security flaws are increasing, and they depend on the architectural layer in which they are implemented. In this context, IoRT connectivity management and platforms orchestration will play a critical role in addressing the security of IoRT applications in the future.

IoRT applications are addressing massive and critical segments of 5G capabilities. For safety-critical IoRT, Ultra-Reliable Low-Latency Communications (URLLC) architecture and radio are required. Mission-critical IoRT applications will benefit from the new 5G core network architecture, as well as from enhancements in the radio. The capabilities of 5G networks are built upon 4G Long-Term Evolution (LTE) networks, along with NarrowBand-IoT (NB-IoT) and LTE category M1 (LTE-M or LTE for Machines). The new, advanced functions for security, automation, and management functions on radios and 5G core networks fit the ultra-reliability requirements for safety- and mission-critical IoRT solutions. Considering that IoRT use cases and applications are diversifying along with IIoT, consumer IoT, and enterprise IoT applications, connectivity across vertical domains will be ensured by mobile cellular and enhanced wireless technologies. The new 5G NR in unlicensed spectrum (NR-U) is important for future IoRT applications as the cellular connectivity and of 5G is brought to unlicensed spectrum. NR-U supports both license-assisted and standalone use of unlicensed spectrum and can deliver improved coverage capacity, mobility, reliability, and precise timing. For time-sensitive, safety-critical IoRT applications it is important to emphasize that 5G can be integrated with time-sensitive networking (TSN), providing deterministic services over IEEE standard 802.3 Ethernet wired networks, delivering low-latency packet transport, low packet delay variation and low packet loss.

The next-generation Internet is evolving, and although the characteristics have been defined, there is no defined architecture for it. The next-generation Internet must be human-centric, and this foundation creates different requirements for any Internet system to be more adaptive to humans and human-machine interactions than to technology.

IoRT applications' connectivity technologies need further development to meet the high demands for ultra-high reliability, very low-latency, high-bandwidth signal quality, and data rates. These elements are required for safety in many IoRT applications, depending on the context in which the IoRT technologies are applied. In this context, safety and mission-critical IoRT capabilities will not be widely available before 2025–2030.

IoRT applications must consider the interference among different small cells, and the radiation as the density of 5G cells deployed is increasing. At the same time, different network management models must be developed to control roaming, considering the coexistence of various cells, radio access technologies and devices. For areas that are remote or difficult to access, satellite communications are a future radio-access technique for IoRT applications operating in remote environments.

A trade-off between the criticality of the IoRT application, the radio access technique, edge and cloud processing, and cost must be made to minimize latency.

The deployment of 5G networks and beyond requires the densification of the mobile network, which increases the number connections to the core network, entailing the implementation of cloud utilization mechanisms to maximize efficiency (e.g., latency, security, energy efficiency, accessibility). The cloud utilization mechanisms combine SDN and NFV techniques to ensure network flexibility, integrate new applications dynamically, and appropriately configure network resources (e.g., sharing edge-cloud computing resources, orchestration edge-cloud computing resources, splitting data traffic, enforcing security rules, implementing QoS parameters, and ensuring mobility). In this context, the important challenges are (1) designing system and network architectures to easily support efficient and diverse services, (2) achieving ultra-low latency communications with distributed edge computations, (3) defining and managing edge/fog systems, and (4) migrating the Internet edge to computation-enabled intelligent robotic devices at the edge.

IoRT devices are connected via different categories of wireless technologies: short to mid-range wireless (e.g., Bluetooth, Bluetooth mesh, and other mesh networking such as Wi-Fi mesh, ZigBee, Thread/6LoWPAN, etc.), long-range wireless [e.g., mobile/cellular and Low-Power Wide-Area Networking (LPWAN)], and satellites. Expanding IoRT applications will require extending the spectrum in the 10–100 GHz range and unlicensed bands and technologies, such as WiGig or 802.11ax, to deploy IoRT technologies. IoRT connectivity technology classes can be further divided into short-range and wide-area connectivity segments, with the former enabled by unlicensed radio technologies (e.g., LoRa, Sigfox, On-Ramp Wireless, NWave/Weightless SIG, 802.11 Wi-Fi/Wi-Fi Aware, Bluetooth, ZigBee, 6LowPAN, Z-Wave, EnOcean, Thread, and wireless M-Bus), and the simultaneous use of multiple industrial, scientific and medical (ISM) radio bands (i.e., 169/433/868/915 MHz, 2.4, 5.8, and 60 GHz). The load of different networks differs; some models use the unbalanced load of the *ad-hoc* network from a core network standpoint, while others use network-based solutions to balance the topology. The network requirements to be supported are based on a combination between an optimal *ad-hoc* network topology, the use of monitoring information, and the notification of appropriate actions (Vermesan et al., 2018).

Using intelligent robotic devices reduces the amount of data needed to be transferred to the cloud for filtering, processing, aggregation, allowing more functions and processing capabilities to be integrated into IoRT devices and gateways closer to the edge, eliminating the need for extra communication and reducing latency while optimizing the processing of information and communication. Various edge computing paradigms are used for IoRT applications. They are classified as edge computing, fog computing, and Mobile Edge Computing (MEC). MEC is part of the 5G architecture, enables an open Radio Access Network (RAN), and is capable of hosting third-party applications, functions, services, and content at the edge of the network. In edge computing, the intelligence and power of the edge processing unit are defined by programmable automation controllers, with each edge device operating independently and defining what information can be stored locally and what information is transferred to the cloud for more analysis.

IoRT applications are expected to require more network intelligence residing closer to the robotic devices by using edge, fog, MEC-distributed architectures, and cloud/edge federated solutions, considering that information is generated and used locally. Developing edge computing technologies for IoRT requires addressing the issues of unreliable and intermittent information transfer via wireless and mobile cellular networks, efficient distribution of computing processing and analytics, the orchestration and management of data storage and processing, interfacing/orchestration between edge and cloud computing to ensure scalable services, and finally, reliable, robust, and resilient mechanisms to secure IoRT applications. The different edge computing models require distributed architectures that support the various exchange of information and communication protocols for broad use in consumer/business/industrial domains. To implement this, “it needs to provide peer-to-peer networking, edge-device collaboration (e.g., self-organization, self-awareness and self-healing), distributed queries across data stored in edge devices, as well as in the cloud and temporary storage locations, distributed data management (e.g., for defining where, what, when and how long, in relation to data storage) and information governance (e.g., information quality, discovery, usability, privacy and security)” (Vermesan et al., 2018).

Multi-access edge computing performs an important role in IoRT functionality as reliable low-latency processing and information transfer is required for most IoRT use cases. Processing information created within the network and by robotic devices at the edge is enabling computations to be performed on distributed device nodes rather than in a centralized cloud environment reducing the overall latency and improving the overall IoRT system performance.

In addition, for IoRT applications, edge intelligence is moving from the cloud to the robotic things with more DL, ML, and other AI-based computation transferred from the cloud to the edge thereby assuring different distributed, low-latency and reliable, intelligent services. The advantages of decentralizing and distributing the architecture for DL techniques can include the following elements (Kang et al., 2017; Han et al., 2019):

- DL services are deployed at the edge close to service requests, and cloud processing is used when additional computing is required, reducing the latency and cost of transferring data to the cloud for analytics.
- The raw information needed for DL services is stored locally on the edge devices, robotic things, edge infrastructure and not in the cloud, ensuring faster access and better data protection.
- The decentralized architecture could provide more reliable and efficient real-time DL computation.
- IoRT devices are using smart data and application scenarios that edge computing can promote the ubiquitous application of DL and support providing AI for each robotic thing, person and application everywhere.
- The use of various, diversified and valuable DL services for IoRT devices can expand the value of edge computing and accelerate its acceptance, deployment, and growth.

## IoRT Platforms

An IoRT platform can be defined as an intelligent application layer that connects robotic things to network infrastructure and abstract applications to robotic things, enabling communication, information flow, device management, and various functionalities in order to support the development and deployment of intelligent applications and services. The IoRT platforms provide flexibility (the ability to deploy things in different contexts), scalability, usability (the ability to make users' experience easier), interoperability, integrability, and different degrees of intelligence, with the overall aim to build IoRT applications within a framework that allows applications to connect IoRT devices, applications and people to information and control centers (Vermesan et al., 2017b).

Different types of IoRT platforms have emerged, and the functionality of these platforms covers the digital value chain of an end-to-end IoRT system, from sensors/actuators, processing hardware, connectivity, edge computing, storage, cloud, and applications. The connectivity platforms are used to connect edge devices, process information at the edge and program devices to make decisions in real-time. The significant advantages are increased security, interoperability, scalability, and manageability, which are achieved through advanced information management and analytics from an endpoint to edge infrastructure.

IoRT software platforms (e.g., HW/SW robot platforms, IoT/IIoT/DLT platforms, and AI platforms) integrate heterogeneous sensors/actuators, as well as various communication protocols with abstract complexities, and support developers with simple Application Programming Interfaces (APIs) to communicate with IoRT devices over a connectivity network. IoRT platforms include functional components for information collection, processing, storage, and analytics, managing complex information and event integration, protocol conversions, connectivity issues, and device management and orchestration. The platforms provide a frame of reference for categorizing the capabilities of technologies that are needed to deliver connected robotic device functionalities, operations, assets and integration into other platforms (e.g., enterprise platforms).

IoRT platforms provide the components used for the implementation of the IoRT-layered architecture and include the following elements (Gluhak et al., 2016; Vermesan et al., 2018):

- Physical IoRT devices and resources are abstracted into virtual entities and representations, which allows interoperability based on consistent access to heterogeneous IoRT edge devices and their resources via multiple communication protocols.
- Virtualization facilitates service look-up mechanisms that link the physical network's edge and provide virtualization services for different IoRT functions.
- The data management framework enables collected data from IoRT devices to be stored, cached and queried, and data fusion and event management to be performed for IoRT devices while considering scalability and manageability.
- The semantic representation (Honti and Abonyi, 2019) allows the modeling and administration of semantic knowledge from different IoRT devices and platforms.
- The security and policy framework implements the federated identity management for authentication, authorization policies, the access control mechanisms, and facilitates the exchange and coordination among several IoRT platforms.
- The networking framework enables connectivity within and among IoRT and IoT/IIoT platforms, ensuring the instruments for self-management (i.e., monitoring, configuration, healing, optimization, and protection) through cognitive algorithms.
- Open interfaces are a set of open APIs that support IoRT applications and facilitate platform extension by allowing the interaction with other platforms, and the development of cross-platform tools for API on top of the platform.
- Data analytics utility provide real-time event analytics and a self-service rule engine to support developers to define rules, as well as querying, reporting, and data visualization functionalities.
- ML data analytics involves a set of complex ML algorithms for real-time cognition and decision-making.
- Development tools, and standardized software development kits (SDKs) provided to accelerate the rapid development of IoRT applications that can be adopted by different applications.

**Figure 11:**
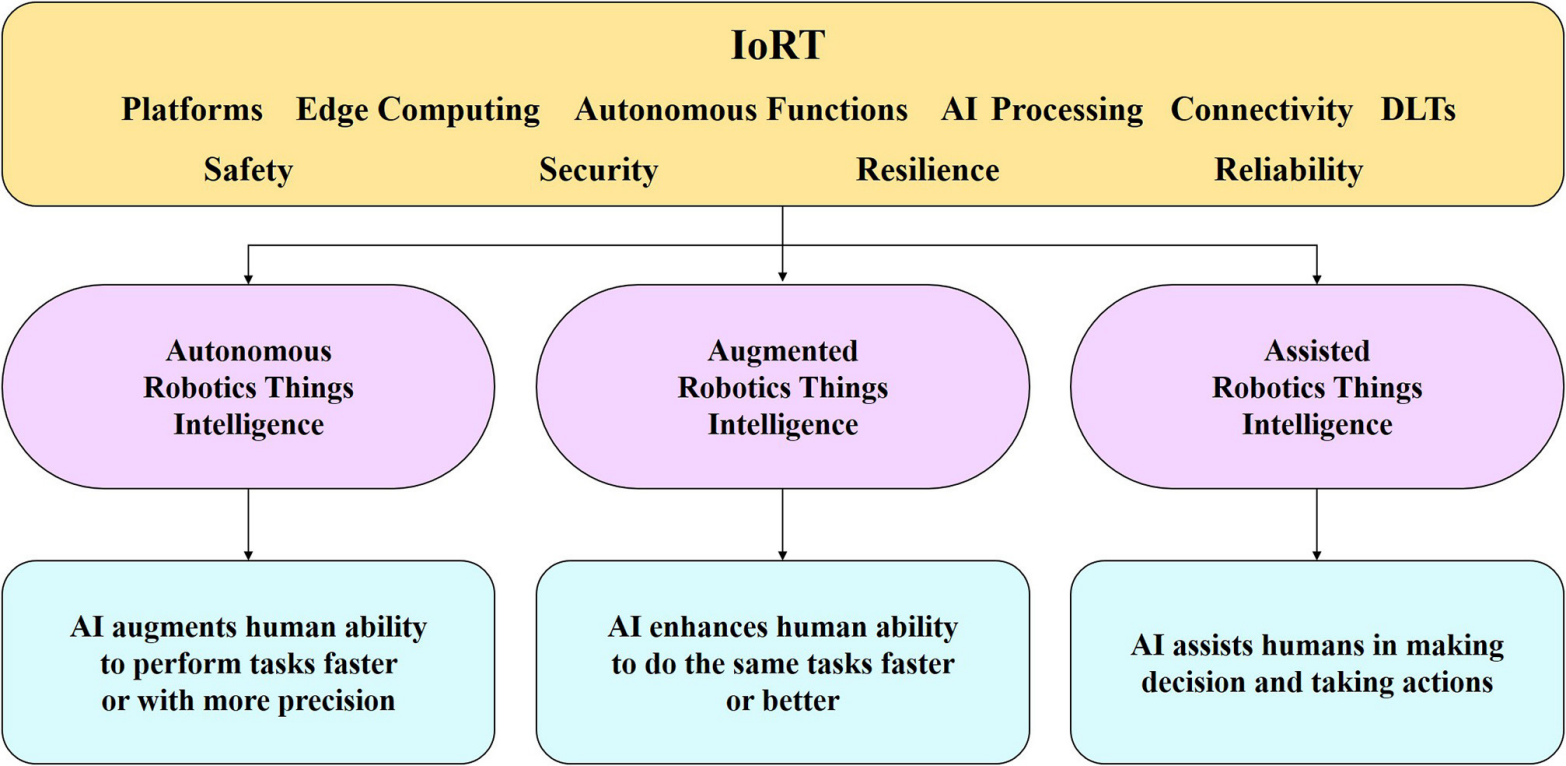
Capabilities implemented at the edge for autonomous/augmented/assisted intelligent functions.

The development of AI methods and techniques that enhance robotic device capabilities are implemented on edge for autonomous/augmented/assisted intelligent functions as illustrated in Figure 11.

This approach requires IoRT platforms to be able to deal with distributed intelligence (i.e., AI on edge and in the cloud) and move from a centralized intelligence architecture (i.e., AI in the cloud).

- Information flow and exchange across the layers of the IoRT architecture involve information acquisition, information transmission/collection, processing, storage, filtering, analysis/analytics, integration, discovery, usage, exposure (openness), and monetization (Vermesan et al., 2017b):
- Information acquisition involves the gathering and formatting IoRT information before it is passed through different channels/pipelines for ingestion and processing. Information acquisition is challenging for IoRT device due to the various channels used and the need to a proper orchestration of the information fusion at the device, edge, and cloud level.
- Information transmission/ingestion involves the connectivity channels and pipelines used for transmitting IoT information and the ingestion of information to enable the reliable operation of IoRT platforms with various file formats and network connections while considering information volume, data rates, and network neutrality.
- Information processing addresses the processing and fusion of IoRT information from different sources (e.g., sensors, actuators, processes, and virtual devices), and transformation of this information into formats that allow reuse or facilitate immediate action based on real-time events and interactions.
- Information storage addresses the techniques of distributed IoT information storage in which the chosen format or database technology is determined by the type of application safety- mission-critical and the nature of other stages in the information value chain (e.g., analysis, analytics, and the nature of applications). For IoRT applications, it is required that the information is stored and managed in a scalable manner that satisfies the needs of the application that requires fast access to raw or processed information.
- Information filtering uses the active management of IoRT information over the information lifecycle to ensure that information quality requirements are met and, the information can be used across different industrial sectors. IoRT data filtering processes include, among others, content creation, selection, classification, transformation, validation, and preservation.
- Information analysis/analytics addresses every layer of the IoRT architecture and every step in the information value chain. This allows the generation of new insights and actions based on IoRT information from different sources, used across applications. Data analysis transforms raw information into smart insights that can be used in decision-making, as well as for domain-specific purposes. Through exploration and modeling, relevant “smart” data is extracted, and useful “invisible” information with high potential from an IoRT application perspective can be synthesized and extracted.
- Information integration blends a variety of IoRT information sources to provide new insights. It is an important element of any IoRT application.
- Information discovery addresses localization and identification of IoRT information sources, services and evaluation of these sources' attributes, relevance, quality, integrity, security, privacy, cost, coverage, etc.
- Information usage addresses IoRT knowledge-driven applications that need access to IoRT information, methods of analyzing the information, and the development tools and IoRT platforms needed to integrate information analysis into various IoRT applications and use cases. The efficient usage of IoRT information enhances the effectiveness of decision-making by, reducing costs, increasing the added value, and offering portability across applications.
- Information exposure (data sharing) addresses exposure of IoRT information to other IoRT applications, enabling co-creation of value from IoT/IIoT/IoRT information obtained from different heterogeneous edge and platform sources.

### Commercial IoRT Platforms

The IoRT platform market represents a new segment characterized by a complex landscape building in many cases on existing IoT/IIoT platforms. The IoRT applications cover various sectors and, require platforms that offer solutions at the intersection of IT and OT, including dependable, interoperable, and scalable features for integrating heterogenous IoRT devices and fleets operating across different platforms. In this context, the emergence of several blockchain platforms that can enhance the features of IoRT platforms brings challenges for IoRT applications related to the solutions and the interoperability characteristics provided considering the multitude of blockchain platforms custom-made for specific purposes, (e.g., public, private, or consortium), that ads an overhead to manage workflows.

Amazon Web Service RoboMaker (AWS RoboMaker) is an example of a commercial proprietary IoRT platform that provides the features, micro-services to be used for data processing, storage, orchestration of the deployment and operations of fleets of IoRT devices [e.g., autonomous mobile robots (AMR), autonomous ground vehicle (AGV), etc.] used for commercial logistics and consumer cleaning, delivery, and companionship. The platform offers the capabilities to combine the integration of technologies at the robot level (e.g., image recognition, sensing, artificial intelligence, machine learning, reinforcement learning, connectivity, etc.) enabled by a Robot Operating System (ROS) with the ability to navigate, communicate, comprehend, stream data, learn, and collaborate at the platform level with other robotic things. AWS RoboMaker is a proprietary cloud-based solution that provides the tools to simulate, test, and securely deploy robotic applications at scale, providing an integrated development environment (IDE), fleet management capabilities, ROS extensions, and integration with different Amazon and AWS services.

Other examples of companies that are providing commercial platforms for managing fleets of robots are Format, Freedom Robotics, InOrbit, Roco, KUKA (e.g., Navigation and Mobile platforms), OTTO Material Movement Platform, BrainOS, TIAGo Base. The platforms offer cloud-based centralized solutions to build, test, deploy, automate the operations of fleets of robotic things, providing secure services, remote operations, local network access for offline operations and limited integration with other IoRT systems.

The new IoRT ecosystems developments will be influenced in the near future by robot developers that move up in the value chain and become solution providers, IoT/IIoT and cloud platform providers focused on HW/SW, connectivity solutions and large computing infrastructures service providers such as Microsoft Azure, Amazon Web Services (AWS), and Google Cloud.

## Interoperability in IoRT

**Figure 12:**
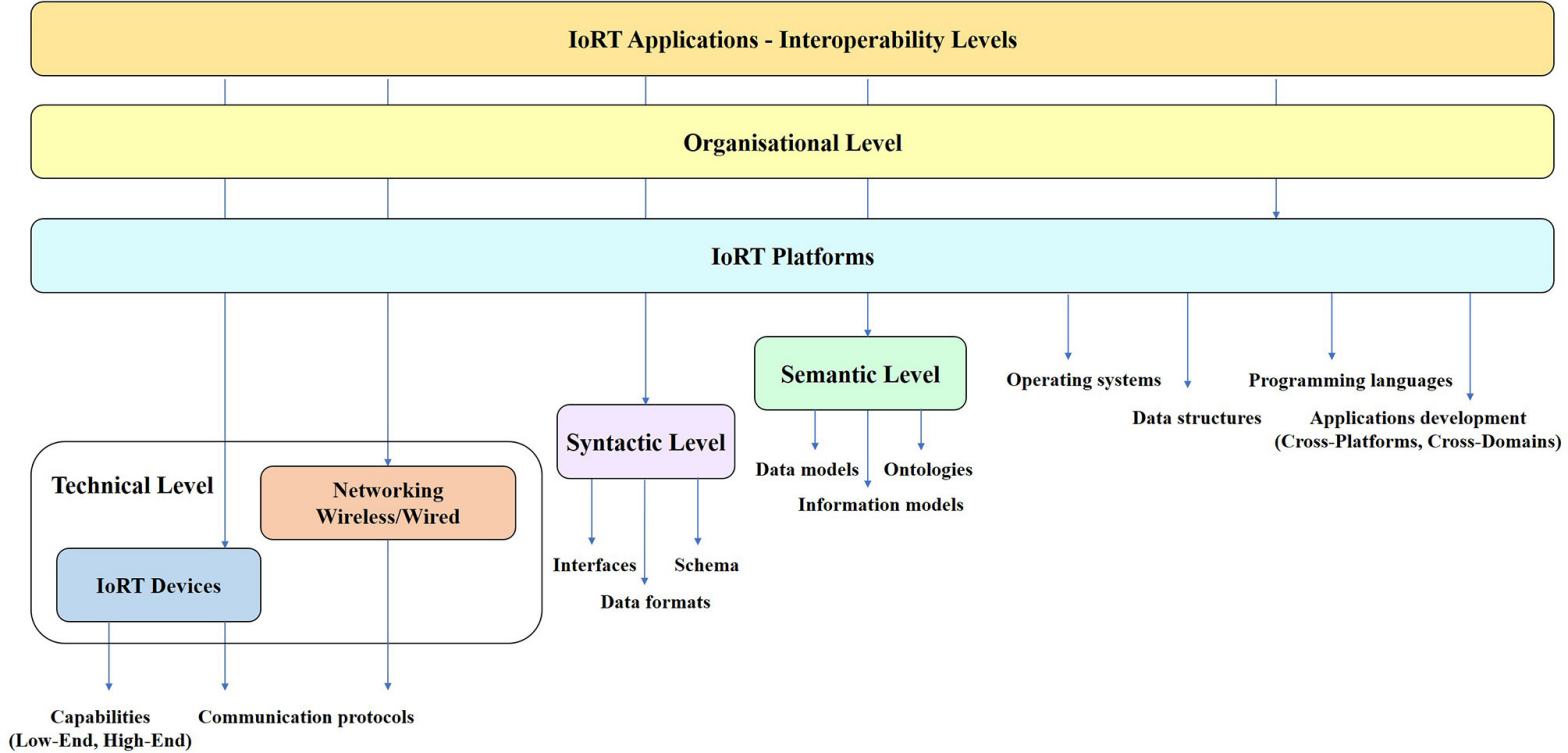
Interoperability levels addressed for IoRT technologies.

IoRT platforms are used to support and manage the development and operation of IoRT applications and services by providing a set of components with the specific functionalities needed to run and orchestrate IoRT applications. IoRT platforms allow developers and service providers to build on a set of components and building blocks that are common and repeatable across different IoRT applications and services, supporting achieving an economy of scale and reducing the overall time and costs required to deliver an IoRT-enabled solution. A lack of platform interoperability gives rise to considerable technological and economic disadvantages, such as the difficulty of plugging non-interoperable IoRT devices into heterogeneous IoRT platforms or developing IoT applications exploiting multiple platforms, stagnation of IoRT technology adoption on large-scale, slow user acceptance of IoRT technology, vertical silos in IoRT ecosystems and markets, increased costs, scarce reusability of technical solutions, and user dissatisfaction (Bröring et al., 2017, 2018; Schmid et al., 2017; Mahieu et al., 2019). The interoperability levels addressed by IoRT technologies are illustrated in Figure 12.

The integration and interaction of heterogenous IoRT systems, require addressing the complex interoperability challenges to facilitate the creation of cross-domain services with seamless movement of physical things and the information that they exchange, store and process. Today, the lack of stable IoRT implementations, a variety of available concepts for implementation, lack of common data formats, data models and the lack of a common IoRT reference architecture, limit IoRT systems interoperability. Another challenge is the lack of a common framework for verification, validation, testing, and certification of different IoRT implementations based on agreed performance requirements.

The interoperability testing practices require various vendors, developers, and service providers to participate in developing and supporting such common frameworks.

Improving interoperability is critical to the success of IoRT applications and enabling the use of interoperability frameworks while federating and connecting several platforms to scale up IoRT applications is key to further developments.

Interoperability testing of solutions and standards to achieve different types of interoperability for heterogeneous and complex systems is a challenge. This is mainly because testing the IoRT solutions involves various stakeholders (e.g., HW/SW vendors, platforms providers, developers, integrators, and service providers) that need to participate several times in face-to-face meetings (e.g., plug tests, to validate their implementation against existing standards). The process is labor-intensive and involves extensive testing activities. Interoperability testing needs to be automated to support the rapid development of interoperable solutions (Noura et al., 2019). In the case of IoRT, new concepts need to be developed, including the virtual validation of autonomous systems and scenarios for collaborative robotic devices.

IoRT platforms provide a diverse set of functional components implemented in the different architectural layers that contribute to the realization of the IoRT service patterns described in the previous section.

The functional components of typical IoRT platforms are briefly described below:

- Robotic device management and orchestration ensure that robotic devices work properly with the IoRT platform and are up to date with the latest firmware/software and security patches. IoRT platforms offer device registration/discovery, device directory/catalog services with capability descriptions, device status monitoring, and tools for over-the-air updating of the devices' firmware and application software. Intelligent robotic devices can update their functions while charging or parking.
- Processing and action management are functions that operate on top of IoRT information streams received from different IoRT devices. This allows for mapping of low-level perception events to higher-level events through logical constructs or/and rules and linking these to new events or action commands to IoRT devices. They can include functions for sensor fusion based on input from the perception sensors of different IoRT devices.
- Information storage is a critical service of IoRT platforms. It aggregates information originating from robotic devices for online or offline processing and other state information that may be related to the devices, including learning/training. Depending on the architecture, edge/cloud storage technologies are used to achieve scalability. Distributed memory and storage are important elements of training/learning algorithms used by different IoRT functions.
- The analytics component includes a collection of tools that enables the extraction of insights from data and the performance of more complex data processing at the edge or in the cloud. These tools range from specific data mining or ML/DL techniques to more AI-based specialized algorithms for different application domains. Offline techniques are used for databases of historical data. Online/offline techniques enable online stream processing across incoming information streams from IoRT devices. IoRT platforms can offer third parties the ability to integrate analytics components.
- External interfaces are APIs used to develop applications and services on top of platform functions. This category also includes development tools or wrappers that can be integrated into other enterprise backend systems.

**Figure 13:**
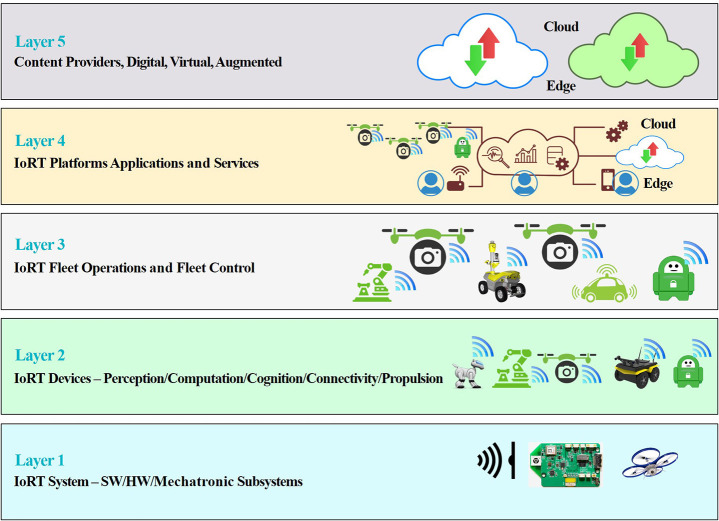
Stack of layers covered by the IoRT functional components.

The full stack of layers covered by the IoRT functional components is illustrated in Figure 13. The illustration shows the case of IoRT applications that cover five layers of the 3D layered IoRT reference architecture.

Extensive techniques and methods for addressing IoT platforms' interoperability that can apply to IoRT platforms are presented in Bröring et al. (2017, 2018). The work covers the interoperability aspects, challenges and approaches that deal with interoperability in existing IoRT platforms and presenting insights regarding future challenges for IoT/IIoT/IoRT platforms' interoperability, achievable solutions, and a potential IoRT interoperability platform architecture.

## Trustworthiness in IoRT

In IoRT applications, the intelligent robotic things devices are enforced with the computation, cognition, learning and connectivity capabilities. In this context, it is important to address how the computations (e.g., information analytics, security, AI, machine learning and blockchain), are seamlessly embedded into the edge infrastructure and robotic things, and how they are enabled to be efficient, trustworthy and accountable when performing safely and reliably, different tasks and operating in different environments.

The current approach used by cloud robotics and AI is strongly related to the availability and elaboration of large amounts of data that is available in the cloud. This approach relies on huge quantities of data that are transferred to the cloud with significant energy that is necessary to run the super-computers that process these data and information. The development of the IoRT applications requires a paradigm shift from centralized processing in the cloud to decentralized, federated cloud-edge processing and, in the future, to distributed processing, cognition, and connectivity at the edge.

The distributed approach could allow processing, cognition and learning to be done at the edge of the cloud, particularly near the sensing and actuation devices that are close to the user. For IoT/IIoT robotic things equipped with voice-activated systems, a significant part of the processing can be done around the sensor, with voice recognition at the level of the robotic thing keeping the data under control, reducing the energy consumption for connectivity and elaboration—while also improving resilience against network failures—and assuring that the elaboration is more accurate for the specific IoRT application's needs. In this way, the IoRT can evolve into an intelligent networked infrastructure of collaborative, artificially intelligent robotic things that interact and collaborate with humans and their natural intelligence.

The requirements for IoRT open platforms to ensure capabilities are accessible across different industrial sectors and to other autonomous systems implies addressing the implementation of a reliable, secure and trustworthy development framework that safeguards that globally-connected, open and interoperable environments can be created by using industry-driven, standards-based solutions.

**Figure 14:**
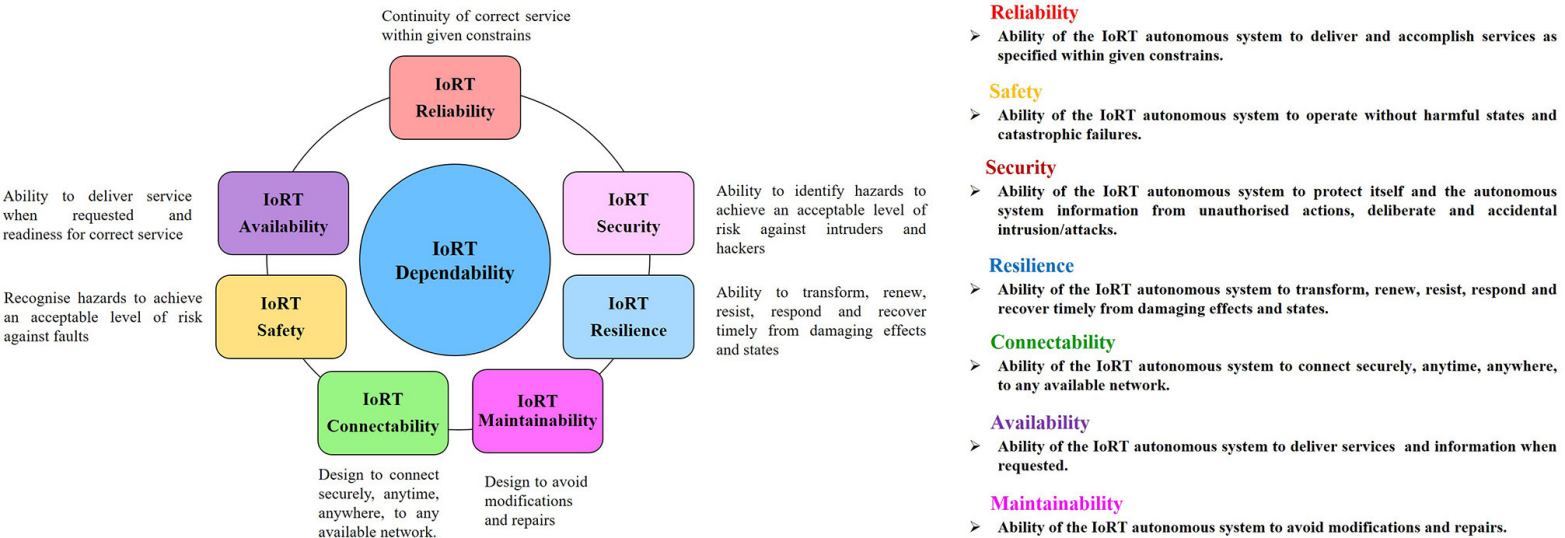
Dependability components for assuring IoRT trustworthiness.

In this context, the trustworthiness of IoRT technologies and applications are directly connected to the concept of dependability. Assuring dependability is providing the basis for trust in IoRT technologies. The dependability components are illustrated in Figure 14.

The trustworthiness of IoRT technologies and applications is key to the adoption of the technology, and many ethical issues must be addressed along the way to develop these technologies. As IoRT applications interact, cooperate and collaborate with humans, addressing ethical issues, such as cognitive biases that affect the algorithms that are used for data mining, interpretation and the decisions of the robotic things, is critical to advancing the development of human-centered IoRT technologies. The new generation of IoRT systems and the underlying AI methods and techniques will need to apply concepts of machine-to-machine and human-to-machine orchestration; otherwise, they are bound to either fail in their scope or experience severe threats to their usage.

## IoRT Applications

The IoRT enables the transition to a digital, hyperconnected society in which every “thing” can sense its surroundings and environment, exchange information, provide feedback, or initiate actions. This is implemented using sensing, processing, cognitive, connectivity, and AI analytics processes at the edge of the network integrated as part of a distributed architecture. In this context, the main benefits of IoRT systems are connected to the network effects that arise when different heterogeneous autonomous systems are integrated, fleets of IoRT devices are interacting and used in different application areas to provide new services.

The novelty of the concept of IoRT extends to multiple application areas that demonstrate the convergence of various technologies integrating collaborative, heterogeneous intelligent robotic things, and autonomous devices into a distributed reference architecture of knowledge-centered platforms that operate over a computing continuum from edge to cloud and to high-performance computing infrastructure.

IoRT uses the convergence of IoT/IIoT technologies and robotics to enhance robotic capabilities, enabling the aggregation of advanced IoT/IIoT functionalities into novel applications and the development of new business models that increase the investment opportunities. AI techniques enable IoRT cognitive systems to be integrated with IoT/IIoT applications to create optimized solutions and fleet-based services and applications.

There are numerous applications for IoRT fleets, and the major benefit of digitalization is the ability to analyse and optimize machine performance in real-time, using data generated by embedded sensors. An example of an application for the IoRT fleets is the concept of self-maintenance of the fleet based on the real-time information, using predictive maintenance models, digital simulation, and identification of trends to provide maintenance information based on actual usage and wear characteristics of the IoRT devices. Companies such as ABB have already developed and deployed solutions like Fleet Assessment to benchmark the connected robots, provide preventive care and condition monitoring/diagnostics.

The IoRT applications integrate different types of devices such as collaborative robotic things mobile robotic things (e.g., automated guided vehicles—AGVs), lightweight mobile platforms, used as fleets in warehouses and distribution centers, manufacturing intralogistics, agriculture, and specific environments in logistics in hospitals or retail.

The fleets of robotic things (service and humanoid robots) are used for logistics and delivery as well as for moving objects, such as boxes, pallets, or tools, in industrial settings between machinery, transfer points, or storage areas.

The IoRT applications are expanding in the field of professional service robotic things, personal service healthcare, defense, rescue, security, logistics, construction, agriculture, professional cleaning, inspection, and maintenance, domestic, entertainment and leisure.

The current pandemic crisis has shown the need to accelerate the developments of IoRT technologies and applications for deploying fleets of robotic things for healthcare assistants, logistics, delivery of goods using autonomous robotic things.

IoRT technologies and applications can be deployed in various industries including healthcare, defence, security, agriculture, forestry, logistics, construction, professional cleaning, domestic, and entertainment.

An overview of application areas for IoRT is given in Grieco et al. (2014), Vermesan et al. (2017b) Duckett et al. (2018), Zachiotis et al. (2018), Ramdani et al. (2019), Tractica (2019), Sitaramanjaneya et al. (2019), Guizzo (2020), and Galar et al. (2020).

Many IoRT applications are at early stages with different companies and research groups testing various solutions as IoRT deployments could provide many advantages such as reduced costs of services, speed, reduction in pollution, etc.

The acceptance of IoRT technologies and applications depend on the features provided concerning enhanced usability, delivery of accurate and high-quality services, reliability, reduced operational costs, safety, and machine errors.

The high upfront investment, the concerns for human safety could limit the IoRT market growth in the short term. The lack of common standardization and regulation framework, lack of interoperability, rules for the coexistence of IoRT fleets in different environments or public urban areas, congestion, the potential for theft, injury, and blocking access to other mobility means are seen as obstacles to the adoption of the IoRT technologies, and new solutions are expected to properly address these issues.

### Humans—Robotics Things Interaction and Collaboration

The deployments of IoRT fleets in industrial sectors to replace, assist and support humans to perform different tasks require the development of new methods and techniques for humans—robotics things interaction and collaboration in conditions and environments where humans and robotics things are sharing the same workspace, and they are co-workers collaborating to accomplish different tasks in industrial environments. An overview of human-robot interaction systems that can utilize the capabilities of both humans and robots is presented in Hentout et al. (2019). The article presents a literature review of major works on human-robot interactions in industrial collaborative robots, conducted during 2008 and 2017 and proposes a classification of the content of these works into several categories and sub-categories.

Makris et al. (2018) and Michalos (2017) presented several IoT and industrial robotics case studies and challenges related to humans and robotics things interactions with the example from different implementation scenarios.

Müller et al. (2017) proposed a classification for the different ways in which humans and robotic things can create a collaborative environment through coexistence (e.g., the human operators and robotic things are in the same environment and do not interact with each other), synchronized (e.g., the human operators and robotic things work in the same workspace, at different time slots), cooperation (e.g., the human operators and robotic things work in the same workspace/environment at the same time slot, each focusing on separate tasks), collaboration (e.g., the human operators and the robotic things must work on a task together with immediate consequences of the actions of one on the other, by using specific sensors/actuators and vision systems).

An extensive literature review and overview of collaborative robotics toward manufacturing applications are given in Matheson et al. (2019) with focus on human-robot collaboration and the related standards and modes of operation. The authors conclude that human-robot collaboration is a new frontier for robotics, and the human-robot synergy represents an important factor in the industry for improving production in terms of performances and flexibility. To successfully deploy the robotic systems in industrial sectors, they must be proved safe for human operators, easy/intuitive to use, and simple to set up.

## Open Issues and Future Directions of Research

This section addresses several open research and innovation issues and provides recommendations for future research guidelines and directions on different topics related to IoRT technologies and applications.

The IoRT technologies need to address the different requirements in terms of low-latency, high-reliability, system's robustness/stability/resilience, and the heterogeneity of the robotic things in different applications and industrial sectors. In this context, several connectivity technologies are available, and IoRT applications need to utilize multiple paths for the connectivity between the robotic things, infrastructure, edge/cloud platforms to avoid the single point of failure. One future research area is enhancing the reliability and robustness of IoRT applications communication over heterogeneous wireless cellular networks and the development of multi-channel links including different types of communication media (e.g., RF, wireless, optical, sound, voice, etc.). The integration of new beam steering active structure array as part of the IoRT devices and infrastructure and suitable multi-frequency, multi-protocols for IoRT applications to achieve the ultra-low latency with ultra-high reliability are important future research areas.

The IoRT applications need to deal with heterogeneous devices, platforms and address the management of heterogeneous resources including computing, network, storage, and the orchestration between cloud and edge platforms.

The energy efficiency, processing, computational and real-time analytics efficiency of IoRT edge devices, energy-efficient task offloading and intelligent service response time of other IoRT devices and agents, need to be addressed by developing new techniques for collaborative edge-cloud processing and provide dynamic management of network/resource slices, dynamic device management together with the required confinement across different IoRT devices and different complex applications.

More research is needed to understand and find solutions for addressing the trade-offs between the storage vs. link load, memory vs. data rate, capacity vs. latency in IoRT mobile edge, and cloud computing platforms by analyzing the different communication protocols characteristics, location, environmental conditions, wireless link parameters and their impact on end-to-end latency and reliability.

This opens for more work on the dynamic resource allocation across different communication networks (He et al., 2018) by addressing the optimal use of processing resources (processor speed, memory size, computation rate/power), connectivity resources (access bandwidth, fronthaul, backhaul, transmit power, antennas, energy efficiency, power, etc.), memory resources (storage) and learning resources (e.g., AI training and inference).

The use of AR/VR technology for IoRT applications requires new ways of addressing the optimization of the transmission of AR/VR over the dynamic wireless channels for real-time high data-rate and low-latency IoRT applications and how to improve compression, data analytics and processing to support IoRT devices to take better decisions in real-time.

Another open research area is related to online processing of haptic feedback for real-time interactions of IoRT devices, in-field processing to reduce ingress information transfers, and support of processing-intensive AI computation and training at the tactile IoRT edge device that performs safety and mission-critical manipulation and movement tasks. New more efficient ML/deep learning algorithms that implement predictive and extrapolative/interpolative ML solutions are needed for the IoRT devices that operate at the edge of the network to improve the reliability and stability of haptic communications and to enable higher precision and robustness between IoRT tactile edge devices.

Due to the nature of IoRT deployments, the IoRT technologies and applications are prone to security threats that can affect the safe operation of IoRT devices and services. New research is needed to develop secure-by-design and end-to-end security concepts across all the IoRT architectural layers (IoRT devices, connectivity, storage, platforms, etc.). The cybersecurity research priorities for IoRT technologies include communications, authentication techniques, authorization methods, cryptography algorithms, privacy-by-design/by-default, security open-source industrial robotics frameworks, libraries, and tools.

IoRT technology presents a huge challenge to standardization and legal bodies that requires a new approach to standardization, certification, and legislation within a common global policy framework consistent with the standardization work in different enabling technologies domains. The future IoRT standardization activities need to focus on technical standards that address interoperability, functionality and safety aspects of IoRT technologies and applications, based on the needs of industry, regulators, users, in areas such as data format for information exchange between IoRT devices/platforms, security, privacy, validation, testing certification of IoRTs, covering physical/virtual validation, reliability, functional safety, fail-operational, emergency operation of IoRTs, perception of the IoRT's devices external environment, human IoRT devices interfaces, human factors and ethical aspects.

## Summary and Conclusions

Next-generation IoRT embeds more sensing, actuation, cognition, computation, and connectivity components, and they can deal with highly complex and dynamic real-world tasks while cooperating, exchanging information, and collaborating with other robotic things, IoT/IIoT devices and humans.

In this article, we elaborated on the latest concepts related to IoRT, emphasizing the IoRT intelligent connectivity, architectures, interoperability, and trustworthiness framework, and surveying the technologies that enable the application of the IoRT across different domains.

IoRT enabling technologies are summarized into several categories such as IoT/IIoT technologies, autonomous robotic systems, intelligent connectivity, distributed and federated edge/cloud computing, AI, digital twins, DLTs, virtual and augmented reality, swarm and platforms technologies.

The article gives an overview of the IoRT taxonomy, its developments and the emergent architectures and challenges for the integration of intelligent systems and things into applications for collaborative autonomous systems, which require the convergence of technologies such as IoT/IIoT, robotics, AI, intelligent connectivity and trustworthiness frameworks for the deployment of IoRT solutions.

IoRT technologies and applications have developed in the last few years, but as identified in this paper, there are many challenges to be addressed due to convergence of technologies, the existence of different architectures, different underlying robotics technologies that are used, heterogeneity of IoRT devices, various centralized cloud solutions that need to federate to emerging edge infrastructure.

Enabling IoRT interoperability frameworks for connecting several platforms requires solutions that are realistic and scalable to multiple IoRT and data platforms with the possibility to plug and play dynamically new IoRT platforms when a new IoRT application is integrated. The interoperability should be made available regardless of the underlying IoT/IIoT/IoRT, robotics technologies and platforms used.

The connectivity is still a challenge when using multiple communication protocols (e.g., wireless, cellular, and optical) for connecting the IoRT devices. The latency, reliability, robustness security (e.g., authentication, identification, encryption, integrity, etc.) are critical for IoRT applications with new techniques emerging for device management, edge-cloud orchestration, and over-the-air software updates.

## Author Contributions

OV coordinated the structure and elaboration of the paper and the alignment and integration of individual contributions. All authors contributed ideas, content, perspectives, and references based on the work in different common projects and discussed the manuscript.

## Conflict of Interest

OV is employed by the company SINTEF AS, Norway. RB is employed by the company SINTEF AS, Norway. MO is employed by the company Infineon Technologies Austria AG, Austria. MS is employed by National University of Ireland Galway, Ireland. TK, TW, and HS are employed by the company NxTech AS, Norway. MA is employed by the company Infineon Technologies India Pvt. Ltd., India. MG is employed by the company Semiconductors GmbH, Austria. The authors declare that the research was conducted in the absence of any other commercial or financial relationships that could be construed as a potential conflict of interest.

## Footnotes

^1^AutoDrive - Advancing fail-aware, fail-safe, and fail-operational electronic components, systems, and architectures for fully automated driving to make future mobility safer, affordable, and end-user acceptable, Online at: https://autodrive-project.eu

^2^AUTOPILOT - Automated driving progressed by Internet of Things, Online at: https://autopilot-project.eu/

^3^CREATE-IoT - Cross fertilisation through alignment, synchronisation and exchanges for IoT, Online at: www.create-iot.eu/
